# Mycoparasitism related targets of Tmk1 indicate stimulating regulatory functions of this MAP kinase in *Trichoderma atroviride*

**DOI:** 10.1038/s41598-023-47027-6

**Published:** 2023-11-15

**Authors:** Lea Atanasova, Martina Marchetti-Deschmann, Albert Nemes, Bianca Bruckner, Pavel Rehulka, Nancy Stralis-Pavese, Paweł P. Łabaj, David P. Kreil, Susanne Zeilinger

**Affiliations:** 1 Department of Food Science and Technology, University of Natural Resources and Life Sciences Vienna (BOKU), Vienna, Austria; 2 Department of Microbiology, Universität Innsbruck, Innsbruck, Austria; 3 Institute of Chemical Technologies and Analytics, TU Wien (Vienna University of Technology), Vienna, Austria; 4 Department of Molecular Pathology, Faculty of Military Health Sciences, University of Defence, Hradec Králové, Czech Republic; 5 IMBT Bioinformatics, Department of Biotechnology, University of Natural Resources and Life Sciences Vienna (BOKU), Vienna, Austria

**Keywords:** Microbiology, Molecular biology

## Abstract

Mycoparasitism is a key feature of *Trichoderma* (Hypocreales, Ascomycota) biocontrol agents. Recent studies of intracellular signal transduction pathways of the potent mycoparasite *Trichoderma atroviride* revealed the involvement of Tmk1, a mitogen-activated protein kinase (MAPK), in triggering the mycoparasitic response. We previously showed that mutants missing Tmk1 exhibit reduced mycoparasitic activity against several plant pathogenic fungi. In this study, we identified the most robustly regulated targets that were governed by Tmk1 during mycoparasitism using transcriptome and proteome profiling. Tmk1 mainly exerts a stimulating function for *T. atroviride* during its mycoparasitic interaction with the fungal plant pathogen *Rhizoctonia solani,* as reflected by 89% of strongly differently responding genes in the ∆*tmk1* mutant compared to the wild type*.* Specifically, 54% of these genes showed strong downregulation in the response with a deletion of the *tmk1* gene, whereas in the wild type the same genes were strongly upregulated during the interaction with the fungal host. These included the gene encoding the mycoparasitism-related proteinase Prb1; genes involved in signal transduction pathways such as a candidate coding for a conserved 14-3-3 protein, and a gene coding for Tmk2, the *T. atroviride* cell-wall integrity MAP kinase; genes encoding a specific siderophore synthetase, and multiple FAD-dependent oxidoreductases and aminotransferases. Due to the phosphorylating activity of Tmk1, different (phospho-)proteomics approaches were applied and identified proteins associated with cellular metabolism, energy production, protein synthesis and fate, and cell organization. Members of FAD- and NAD/NADP-binding-domain proteins, vesicular trafficking of molecules between cellular organelles, fungal translational, as well as protein folding apparatus were among others found to be phosphorylated by Tmk1 during mycoparasitism. Outstanding downregulation in the response of the ∆*tmk1* mutant to the fungal host compared to the wild type at both the transcriptome and the proteome levels was observed for nitrilase, indicating that its defense and detoxification functions might be greatly dependent on Tmk1 during *T. atroviride* mycoparasitism. An intersection network analysis between the identified transcripts and proteins revealed a strong involvement of Tmk1 in molecular functions with GTPase and oxidoreductase activity. These data suggest that during *T*. *atroviride* mycoparasitism this MAPK mainly governs processes regulating cell responses to extracellular signals and those involved in reactive oxygen stress.

## Introduction

Sensing and responding to environmental changes is crucial for the survival of all living organisms. Most extracellular cues are perceived by membrane-bound receptors, which, upon stimulation by binding of a ligand, relay the information to intracellular signaling pathways. In eukaryotic cells, conserved mitogen-activated protein kinase (MAPK) cascades are involved in transducing a variety of extracellular signals. The core unit typically comprises three serine/threonine protein kinases, a MAPK, a MAPK activator (MAPK kinase, MAPKK), and a MAPKK activator (MAPKK kinase, MAPKKK) among which signal transmission is achieved by sequential phosphorylation^1^. MAPK signaling pathways affect the regulation of gene expression as well as cytoplasmic activities essential in the adaption of the cell to a stimulus and have been shown in the regulation of developmental processes in plants, mammals, insects, slime molds and fungi^2–4^. The budding yeast *Saccharomyces cerevisiae* contains five MAP kinases regulating mating, invasive growth, cell wall integrity, hyperosmoregulation, and ascospore formation^5^. Although the protein modules as well as upstream regulators and downstream substrates are highly conserved, most fungi only have three MAPK cascades including a single homolog for each of the yeast Kss1/Fus3, Slt2, and Hog1 MAPKs^2,6,7^.

The orthologues of Fus3/Kss1 have been studied in several fungi revealing their importance in regulating virulence-associated processes in biologically and taxonomically diverse phytopathogens^2,8^. In the appressoria-forming *Magnaporthe oryzae* (*M. grisea*), the Fus3/Kss1 orthologue Pmk1 (pathogenicity MAPK) is essential for penetration of rice tissue and infectious growth inside the plant^9^ and studies with other phytopathogens including *Fusarium oxysporum, F. graminearum, Ustilago maydis, Cochliobolus heterostrophus,* and *Botrytis cinerea* have indicated the Pmk1 cascade as a well conserved signaling pathway for regulating plant infection (reviewed in Zhao et al.^8^).

However, only little is known about the transcriptional changes elicited by this pathogenicity MAPK (Pmk) pathway and it is still not clear how it exactly regulates virulence of filamentous fungi. Transcriptional profiling of *M. oryzae* wild-type (WT) and the nonpathogenic ∆*pmk1* mutant during appressorium development revealed genes specifically involved in response to exogenous stimuli, transporter-encoding genes and putative transcription factor-encoding genes as being over-represented among the positively regulated Pmk1 targets^10^. In a study comparatively analyzing the gene expression patterns of null mutants of several components of the Pmk MAPK pathway including the MAPKKK Mst11, the MAPKK Mst7, and the MAPK Pmk1, the vast majority of the regulated genes was found to be mutant-specific. The genes regulated in the ∆*mst11*, ∆*mst7* and ∆*pmk1* mutants during appressorium formation mainly showed down-regulation and were associated with metabolism, cell signaling, protein biosynthesis and processing, and transcriptional regulation^11^. Transcriptional analysis of a *Ccpmk1* deletion mutant in the woody plant‐pathogenic fungus *Cytospora chrysosperma* during the simulated plant infection process revealed a downregulation of a series of transcription factor genes and putative effector genes which might be important for fungal pathogenicity^12^.

The fungal genus *Trichoderma* (Hypocreales, Ascomycota) comprises species being potent mycoparasites, i.e. parasites of other fungi, placing them among the best agents for biological control of phytopathogens in agriculture^13^. In the presence of host fungi, differentiation processes are initiated in *Trichoderma* leading to attachment to host hyphae, what is accompanied by an up-regulation of hydrolytic enzymes enabling the mycoparasite to penetrate and degrade the host’s cell wall and utilize its cellular contents^14^. *Trichoderma atroviride* is one of the best studied mycoparasites. Recent comparative genome and transcriptome analyses revealed the presence and host-induced expression of an array of genes involved in the production of secondary metabolites, in the attack and degradation of the host’s cell wall, and genes encoding small secreted cysteine-rich proteins (SSCPs)^15–17^. A pre-requisite for the mycoparasitic attack is sensing and recognizing the host; consequently, the receptors and signaling pathways involved in the activation of the response to the host fungus are of special interest.

In the present study, we have applied transcriptomics and proteomics to dissect the role of the *T. atroviride* Fus3/Kss1-like Tmk1 MAP kinase*.* Previous studies revealed ∆*tmk1* mutants to show unaltered attachment and coiling around host hyphae, to overproduce chitinases, and to have elevated antifungal activity caused by the over-production of low molecular-weight metabolites such as peptaibols and 6-pentyl-α-pyrone^18^. Despite these enhancements, the mutants exhibited reduced mycoparasitic overgrowth and host lysis, suggesting the existence of additional, still unknown genes/proteins that are contributing to the mycoparasitic activity of *T. atroviride*. By using the ∆*tmk1* mutant as tool and by comparing its transcriptome and intracellular proteome both in the absence and presence of the phytopathogen *Rhizoctonia solani* to that of the *T. atroviride* WT strain, we aimed to obtain a global picture of the genes/proteins and processes regulated by the Tmk1 MAPK upon host interaction.

We here present insights into the genes and molecular events being regulated in a Tmk1-dependent manner in the mycoparasite *T. atroviride* upon interaction with a host fungus. Combination with a comprehensive proteomic analysis further allowed us to identify and categorize the dynamic changes of the proteome in response to host contact and to identify mycoparasitism-relevant targets of the Tmk1 MAPK signaling pathway. We found that Tmk1 mainly exerts a stimulating effect during the early contact with the host but also impacts primary metabolism and other signal transduction pathways in *T. atroviride*.

## Results

### The transcriptomic response of *T. atroviride* to the presence of a living host fungus

Gene expression analysis of the *T. atroviride* response to the living host fungus *R. solani* relative to a self-confrontation control (WT-response) revealed 142 differentially expressed genes with a cut-off of |Log_2_FC| > 2. These genes represent candidates with the highest biological changes in their expression levels during the mycoparasitic response of *T. atroviride* to the host fungus. In this gene set, 88 and 54 candidates were found to be up- and downregulated, respectively.

The top ten statistically most robustly regulated genes (aRank in Table S1) comprised a conserved serine/threonine protein kinase, an isochorismatase family protein, GH3 β-glucosidase similar to *T. reesei* CEL3b, translation elongation factor EF2 and an unknown protein, fumarate hydratase, putative pyruvate kinase, RTA1 and RTA1-like proteins, and bifunctional catalase/peroxidase. The isochorismatase enzymes are preferentially found in phytopathogens compared to non-pathogenic ascomycetes^19^ and it was thus speculated that they suppress plant defense by inhibiting salicylic acid formation in plants in response to pathogen attack. In confrontation with *R. solani* this candidate was the most differentially downregulated gene in *T. atroviride* (ninefold-change), whereas the GTP-binding elongation factor 2 was the most upregulated candidate (fourfold-change) in this data set (Table S1).

A closer look on the additional genes with significant differential regulation over the applied threshold during the response of *T. atroviride* WT to a living host fungus regarding putative mycoparasitism-relevant functions revealed candidates that encode proteins involved in sensing, defense, and oxidative stress. Among those, three G protein-coupled receptor (GPCR)-encoding genes were found: a gene coding for a GprK-type GPCR with a RGS-domain was upregulated under mycoparasitic conditions, whereas genes encoding a PTH11-type GPCR carrying a CFEM domain and a putative GCPR of class XIII were found to be downregulated upon contact with the host (Table S1). GPCRs respond to a variety of environmental cues and hence are proposed to be at the sharp end regarding recognition of host-derived signals^14^. Furthermore, several candidates acting in MAPK pathways were found to be upregulated during the mycoparasitic response of *T. atroviride* including Tmk2, a MAPK governing cell wall integrity, and the MAPK kinase Pbs2, which in *S. cerevisiae* is involved in the high osmolarity response pathway^20^. C_2_H_2_ as well as basic-leucine zipper (bZIP) transcription factors were also detected among the genes being regulated in a mycoparasitism-dependent manner. Moreover, candidates encoding enzymes involved in cellular redox reactions including proteins of the alcohol dehydrogenase (ADH) superfamily, FAD-dependent oxidoreductases, a putative GMC oxidoreductase and NADH:flavin oxidoreductase were identified. The high differential expression of a protein containing carbohydrate-binding WSC domains and of SSCP proteins was also detected (Table S1). Hydrolytic enzymes with a putative role in lysis of the host fungus were as well comprised in the differentially regulated gene set and included a putative secreted phospholipase, a GH75 chitosanase, a GH18 chitinase with a CFEM domain present in fungal extracellular membrane proteins, chitinases CHI 18-2 and CHI 18-7, as well as a GH3 β-glucosidase and a GH92 α-1,2-mannosidase. Two detected glutathione S-transferase encoding genes further support the paradigm on their importance in detoxification during the mycoparasitic attack^15^.

### Mycoparasitism-regulated genes whose transcription is governed by *Tmk1*

Gene responses that were transcriptionally dependent on Tmk1 activity were identified by comparing the mycoparasitic response to *R. solani* of the *T. atroviride* WT to that of its ∆*tmk1* mutant. To this end, gene expression of ∆*tmk1* in confrontation with *R. solani* was first compared to that of ∆*tmk1* in confrontation with itself resulting in the ∆*tmk1-*response. Next, the transcriptional response of the ∆*tmk1* mutant to the host fungus was subtracted from the WT-response. When applying the threshold of |aLog_2_FC| > 2, 140 genes remained that represented the candidates with the highest changes in expression response that were governed by Tmk1 during the mycoparasitic interaction with *R. solani* (Table S2). Based on functional (FunCat) predictions a significant number of these most robustly differently responding genes were involved in metabolism, as well as in information pathways, e.g. in processes employing proteins with binding functions or cofactor requirements, and in protein activity regulation (Table S3), often with several of these functions assigned to a protein. Several genes involved in cellular transport, transport facilitation, and transport routes as well as in biogenesis of cellular components were found to be governed by Tmk1 during mycoparasitism. The most robustly implicated processes characterizing the mycoparasitism-dependent response governed by the Tmk1 MAP kinase included specific fungal informational pathways with the strongest support for translation (FunCat ID 12.04), protein binding (16.01), assembly of protein complexes (14.1), regulation (18.01; regulation by binding/dissociation, 18.01.07), ribosome biogenesis (12.01) and aminoacyl-tRNA-synthetases (12.1), and the categories that implicated involvement of mitochondria (42.16), stress response (32.01; unfolded protein response, 32.01.07), and metabolism (respiration, 2.13; and metabolism of energy reserves, 2.19), as detailed in Table S3.

**Figure 1:**
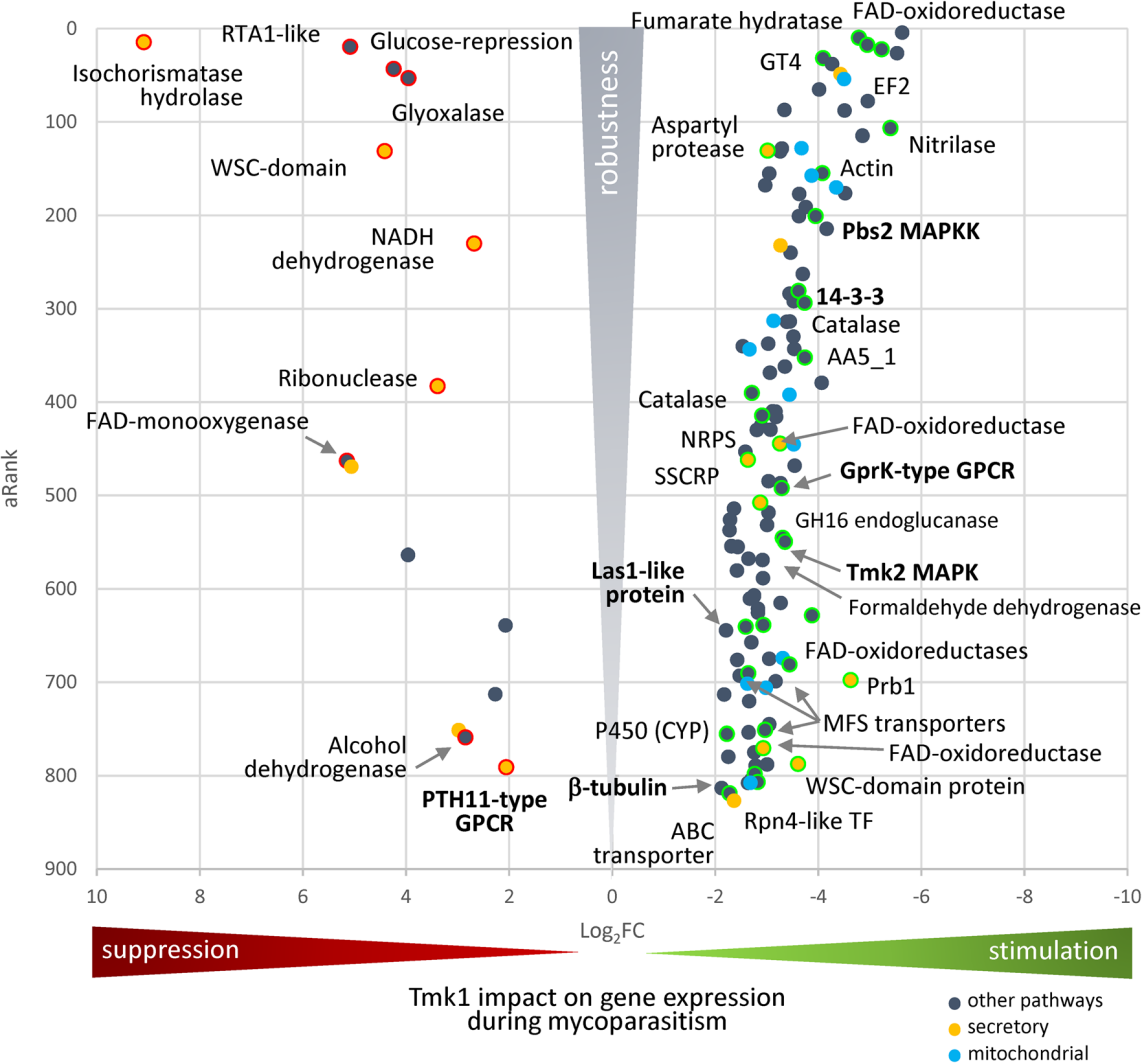
Schematic distribution of statistically significant genes with a strongly different response to the host fungus *R. solani* (|Log_2_FC| ≥ 2) in *T*. *atroviride* ∆*tmk1* compared to the WT. The effect strength is plotted for genes indicating a stimulating role of Tmk1 (green, on the right) or an inhibiting role (red, to the left). The most robust targets governed by Tmk1 during mycoparasitism are towards the left and right edges of the figure. The dot colors represent the involvement in pathways identified for specific genes as shown in the legend, whereas the green and red outline circles represent annotated genes. The names in bold mark the genes involved in signal transduction pathways.

From the total of 140 robustly and significantly differently responding candidate genes, the majority of 66% (93/140) lost in the ∆*tmk1* response to *R. solani* the significant robust upregulation seen in the WT response. A further 22% (31/140) showed a significant robust downregulation in the mutant response that was not seen in the WT. While one gene encoding for an uncharacterized protein was significantly and strongly downregulated in the responses of both mutant and WT, it was significantly and robustly more downregulated in the mutant (ID 227962). Together, these 125 genes (89%) indicate a mainly stimulating function of Tmk1 on gene regulation in *T. atroviride* during mycoparasitism. 54% (68/125) of these genes were strongly regulated in both mutant and WT: 67 were strongly upregulated when the WT was confronted with the host, but showed strong downregulation in the response of the mutant (DOWN.UpWT.DownMut). One gene was significantly and strongly downregulated in the responses of mutant and WT but significantly and robustly more so in the mutant. Among these 67 genes were candidate genes coding for class II fumarases, a FAD-dependent oxidoreductase, an elongation factor 2, and the cross-pathway control-like protein CPC1/Gcn4, which in *Neurospora crassa* coordinates together with histone acetyltransferase GCN5 to regulate catalase-3 expression under oxidative stress^21^ (Table 1). Further this set of 67 genes included genes encoding the mycoparasitism-related proteinase Prb1, genes involved in signal transduction pathways such as a conserved 14-3-3 protein with ability to bind a variety of functionally diverse signaling proteins, including kinases, phosphatases, and transmembrane receptors, and a gene coding for Tmk2, the *T. atroviride* cell-wall integrity MAP kinase, genes encoding a specific siderophore nonribosomal peptide synthetase (NRPS), the Rpn4 transcription factor that stimulates the expression of proteasome genes, actin, and multiple FAD-dependent oxidoreductases and aminotransferases. Besides for the *prb1* gene, a strong stimulatory function of Tmk1 was also detected for genes encoding the following transmembrane proteins: two MFS transporters, a serine palmitoyltransferase, a protein related to urea active transporter, neutral/alkaline non-lysosomal ceramidase, putative cleft lip and palate transmembrane protein 1-like protein, and a hypothetical heavy metal ion homeostasis protein (Table S2). Furthermore, of the 125 genes supporting a stimulating role of Tmk1, 57 were not strongly regulated (regulated below the threshold [Rbt] level; see Table S2) in the response of one strain. In detail, 31 genes were strongly downregulated in the mutant but were not strongly regulated in the WT (DOWN.RbtWT.DownMut), including a GT4 glycosyltransferase and several candidates involved in detoxification processes (Fig. 1, Table S2): a strongly downregulated nitrilase, which might be involved in the hydrolysis of cyanide and nitriles; a glutathione S-transferase domain-containing protein participating in the detoxification of reactive electrophilic compounds by catalyzing their conjugation to glutathione, and several flavine-containing oxidoreductases and putative catalases, several proteins related to pleiotropic drug resistance proteins from the ABC superfamily, and a member of the SSCP family (Fig. 1, Table S2). Conversely, 26 genes were strongly upregulated in the WT but were not strongly regulated in ∆*tmk1* (DOWN.UpWT.RbtMut), including genes coding for pyruvate kinase pykF, aldehyde dehydrogenase, putative aspartyl protease, amidase, but also copper radical oxidase AA5_1, bifunctional catalase/peroxidase, putative metallopeptidases, and a GPCR GprK-type candidate.

**Table 1:** List of the 25 most robustly regulated genes sorted by aggregate rank (aRank) in WT-response *vs* ∆*tmk1*-response to the host fungus. Grey marked genes represent upregulated candidates.

Protein ID	aRank	aLog_2_ FC	Annotation	FunCat number (second rank)	FunCat description
149070	4.37	− 5.63	Hypothetical protein	99	UNCLASSIFIED PROTEINS
297394	9.89	− 4.79	Fumarate hydratase: Class II fumarases	01.05// 02.10// 42.16	METABOLISM//C-compound and carbohydrate metabolism// ENERGY//tricarboxylic-acid pathway (citrate cycle, Krebs cycle, TCA cycle)// BIOGENESIS OF CELLULAR COMPONENTS//mitochondrion
80979	14.65	9.09	Isochorismatase hydrolase	99	UNCLASSIFIED PROTEINS
35737	17.51	− 4.95	FAD-dependent oxidoreductase	01.01//01.07 14.07// 20.01// 42.19	METABOLISM//amino acid metabolism//metabolism of vitamins, cofactors, and prosthetic groups// PROTEIN FATE (folding, modification, destination)//protein modification// CELLULAR TRANSPORT, TRANSPORT FACILITIES AND TRANSPORT ROUTES//transported compounds (substrates)// BIOGENESIS OF CELLULAR COMPONENTS//peroxisome
87275	19.56	5.08	RTA1 like protein	20.01//20.03	CELLULAR TRANSPORT, TRANSPORT FACILITIES AND TRANSPORT ROUTES//transported compounds (substrates)//transport facilities
301275	22.18	− 5.23	Elongation factor 2	12.04// 16.19	PROTEIN SYNTHESIS//translation// PROTEIN WITH BINDING FUNCTION OR COFACTOR REQUIREMENT//nucleotide/nucleoside/nucleobase binding
284578	26.44	− 5.53	Hypothetical protein	99	UNCLASSIFIED PROTEINS
77441	31.62	− 4.09	GT4: glycosyltransferase	01.05// 02.19// 32.01// 34.11// 43.01	METABOLISM//C-compound and carbohydrate metabolism// ENERGY//metabolism of energy reserves (e.g. glycogen, trehalose)// CELL RESCUE, DEFENSE AND VIRULENCE//stress response// INTERACTION WITH THE ENVIRONMENT//cellular sensing and response to external stimulus// CELL TYPE DIFFERENTIATION//fungal/microorganismic cell type differentiation
161217	38.06	− 4.27	Related to cross-pathway control protein 1 CPC1/Gcn4	01.01// 11.02// 16.03// 32.01	METABOLISM//amino acid metabolism// TRANSCRIPTION//RNA synthesis// PROTEIN WITH BINDING FUNCTION OR COFACTOR REQUIREMENT (structural or catalytic)//nucleic acid binding// CELL RESCUE, DEFENSE AND VIRULENCE//stress response
300570	43.30	4.24	Glucose-repressible protein Grg1	99	UNCLASSIFIED PROTEINS
299162	48.97	− 4.43	Serine palmitoyltransferase	01.02//01.06// 01.07// 42.02	METABOLISM//nitrogen, sulfur and selenium metabolism//lipid, fatty acid and isoprenoid metabolism//metabolism of vitamins, cofactors, and prosthetic groups// BIOGENESIS OF CELLULAR COMPONENTS//eukaryotic plasma membrane
37707	53.17	3.95	Putative glyoxalase-like family protein	99	UNCLASSIFIED PROTEINS
133842	54.20	− 4.45	Succinate dehydrogenase/fumarate reductase iron-sulfur protein	01.05// 02.10//02.11// 02.13// 16.01//16.17// 16.21// 20.01	METABOLISM//C-compound and carbohydrate metabolism// ENERGY//tricarboxylic-acid pathway (citrate cycle, Krebs cycle, TCA cycle)//electron transport and membrane-associated energy conservation//respiration// PROTEIN WITH BINDING FUNCTION OR COFACTOR REQUIREMENT (structural or catalytic)//protein binding//metal binding//complex cofactor/cosubstrate/vitamine binding// CELLULAR TRANSPORT, TRANSPORT FACILITIES AND TRANSPORT ROUTES//transported compounds (substrates)
319832	65.24	− 4.02	Probable homocitrate synthase	01.01//01.05	METABOLISM//amino acid metabolism//C-compound and carbohydrate metabolism
161079	77.86	− 4.96	Pyruvate kinase PykF	01.03//01.04//01.05// 02.01// 16.17//	METABOLISM//nucleotide/nucleoside/nucleobase metabolism//phosphate metabolism//C-compound and carbohydrate metabolism// ENERGY//glycolysis and gluconeogenesis// PROTEIN WITH BINDING FUNCTION OR COFACTOR REQUIREMENT (structural or catalytic)//metal binding
301414	87.24	− 3.34	Putative 6-phosphogluconate dehydrogenase	01.05// 02.07// 16.21	METABOLISM//C-compound and carbohydrate metabolism// ENERGY//pentose-phosphate pathway// PROTEIN WITH BINDING FUNCTION OR COFACTOR REQUIREMENT//complex cofactor/cosubstrate/vitamine binding
260330	87.87	− 4.51	Unique *T. atroviride* protein	99	UNCLASSIFIED PROTEINS
154783	106.58	− 5.40	Nitrilase	01.02// 32.05//32.07//36.20	METABOLISM//nitrogen, sulfur and selenium metabolism// CELL RESCUE, DEFENSE AND VIRULENCE//disease, virulence and defense//detoxification// SYSTEMIC INTERACTION WITH THE ENVIRONMENT//plant / fungal specific systemic sensing and response
300828	114.61	− 4.86	Translation elongation factor EF-1 alpha	12.04// 16.01//16.19// 18.02// 40.10	PROTEIN SYNTHESIS//translation// PROTEIN WITH BINDING FUNCTION OR COFACTOR REQUIREMENT (structural or catalytic)//protein binding// REGULATION OF METABOLISM AND PROTEIN FUNCTION//regulation of protein activity// CELL FATE//cell death
260363	128.03	− 3.68	Putative ketol-acid reductoisomerase	01.01	METABOLISM//amino acid metabolism
256374	128.18	− 3.29	Putative aldehyde dehydrogenase	01.05//01.06// 02.11//02.13//02.16// 16.21// 20.01// 32.05//32.07//42.16	METABOLISM//C-compound and carbohydrate metabolism//lipid, fatty acid and isoprenoid metabolism// ENERGY//electron transport and membrane-associated energy conservation//respiration// fermentation// PROTEIN WITH BINDING FUNCTION OR COFACTOR REQUIREMENT//complex cofactor/cosubstrate/vitamine binding// CELLULAR TRANSPORT, TRANSPORT FACILITIES AND TRANSPORT ROUTES//transported compounds (substrates)// CELL RESCUE, DEFENSE AND VIRULENCE//disease, virulence and defense//detoxification// BIOGENESIS OF CELLULAR COMPONENTS//mitochondrion
137844	130.65	− 3.02	Putative aspartyl protease	14.07//14.13// 16.01// 32.01// 34.11// 38.05// 40.10	PROTEIN FATE (folding, modification, destination)//protein modification//protein/peptide degradation //PROTEIN WITH BINDING FUNCTION OR COFACTOR REQUIREMENT (structural or catalytic)//protein binding// CELL RESCUE, DEFENSE AND VIRULENCE//stress response// INTERACTION WITH THE ENVIRONMENT//cellular sensing and response to external stimulus// TRANSPOSABLE ELEMENTS, VIRAL AND PLASMID PROTEINS//viral proteins// CELL FATE//cell death
316963	131.04	4.42	WSC domain-containing protein	01.05//01.25// 32.10	METABOLISM//C-compound and carbohydrate metabolism//extracellular metabolism// CELL RESCUE, DEFENSE AND VIRULENCE//degradation / modification of foreign (exogenous)
302651	131.40	− 3.26	Putative saccharopine dehydrogenase	01.01// 16.21// 32.01	METABOLISM//amino acid metabolism// PROTEIN WITH BINDING FUNCTION OR COFACTOR REQUIREMENT (structural or catalytic)//complex cofactor/cosubstrate/vitamine binding//CELL RESCUE, DEFENSE AND VIRULENCE//stress response
297070	154.66	− 4.08	Actin	10.03//11.02// 14.04//14.07// 16.01//16.07//16.19// 20.01//20.09// 32.01// 40.01// 42.01//42.04// 42.10//42.29// 43.01	CELL CYCLE AND DNA PROCESSING//cell cycle//TRANSCRIPTION//RNA synthesis// PROTEIN FATE (folding, modification, destination)//protein targeting, sorting and translocation//protein modification//PROTEIN WITH BINDING FUNCTION OR COFACTOR REQUIREMENT//protein binding//structural protein binding//nucleotide/nucleoside/nucleobase binding//CELLULAR TRANSPORT, TRANSPORT FACILITIES AND TRANSPORT ROUTES//transported compounds (substrates)//vesicular transport (Golgi network, etc.)// CELL RESCUE, DEFENSE AND VIRULENCE//stress response// CELL FATE//cell growth / morphogenesis// BIOGENESIS OF CELLULAR COMPONENTS//cell wall//cytoskeleton/structural proteins// nucleus//bud / growth tip// CELL TYPE DIFFERENTIATION//fungal/microorganismic cell type differentiation

From the much smaller group of 15 genes (11% of 140) linked to potentially suppressing functions of Tmk1 during mycoparasitism, five genes were strongly downregulated in the WT but were strongly upregulated in ∆*tmk1* (UP.DownWT.UpMut), among which were a RTA1 like protein that may bind to a toxic substance and thus prevent toxicity, a WSC domain-containing protein, a putative glyoxalase/dioxygenase, and a ribonuclease-domain-containing protein (Fig. 1). Six and four genes, respectively, were found to be only strongly down- or upregulated in one of the strains (UP.DownWT.RbtMut and UP.RbtWT.UpMut), as shown in Table S2. Among the genes strongly downregulated in the WT response (UP.DownWT.RbtMut) was isochorismatase hydrolase, which in different organisms catalyzes the conversion of isochorismate into other components, such as 2,3-dihydroxybenzoate and pyruvate and a PTH11-type G protein-coupled receptor. The isochorismatase hydrolase-encoding gene was the highest upregulated candidate found in this study. Isochorismatase hydrolyses isochorismate, which is a very important precursor for salicylic acid biosynthesis in plants. Isochorismatase secretion was shown to be involved in *Verticillium dahliae*’s virulence on potato plants^22^.

Finally, we detected six genes associated with the signal transduction functional category that were governed by Tmk1 during the mycoparasitic interaction with *R. solani*: a PTH11-type GPCR; the Tmk2 cell wall integrity MAP kinase; the Pbs2 MAP kinase kinase being part of the Hog1 pathway in yeasts; a putative member of the 14-3-3 protein family with the potential to bind diverse signaling proteins; a Las1-domain-containing protein with a putative function in the regulation of yeast cell surface growth, bud formation, and morphogenesis; and a putative beta-tubulin characterized by the evolutionarily conserved Tubulin/FtsZ family, GTPase protein domain (Fig. 1). In addition, a putative GPCR related to the RGS domain-containing receptor GprK^23^ from *Aspergillus nidulans* (AN7795) was linked to our candidate genes by sequence similarity search versus the NCBI NR database on 09/07/2020.

In general, a low number of all identified transcripts showed strong stimulation or suppression (17/140 = 12%). Interestingly, almost half (7/15) of the genes suppressed by Tmk1 in the response were predicted to be secreted (Fig. 1, Table S2), a significant enrichment (*p* < 0.2% chi-squared test). Among the secreted proteins were several MFS and other transporters, NADH-ubiquinone oxidoreductase, a serine palmitoyltransferase, neutral/alkaline non-lysosomal ceramidase, metallopeptidase M48, putative PTH11-type and GprK-type GPCRs, and the mycoparasitism-relevant *prb1* proteinase-encoding gene. In addition, 9% (12/140) were found to be involved in mitochondrial pathways; all of them were stimulated by Tmk1 during early mycoparasitism (Fig. 1, Table S2).

### Host fungus-induced proteome remodeling in *T. atroviride* and the role of the Tmk1 MAPK

The protein level can be considered as the level of action in biological systems and, due to splice variations and post-translational modifications, the number of initial target genes is multiplied to a higher number of possible protein candidates interesting for certain biological processes. Consequently, transcriptome analyses were complemented in our study by proteomic approaches for obtaining a more complete understanding of the molecular mechanisms involved in *T. atroviride* mycoparasitism. Mycoparasitism-relevant Tmk1 targets were identified by comparing the proteomes of the *tmk1* mutant and the WT in confrontation with *R. solani* and consequently by identifying proteins with differential abundance.

**Figure 2:**
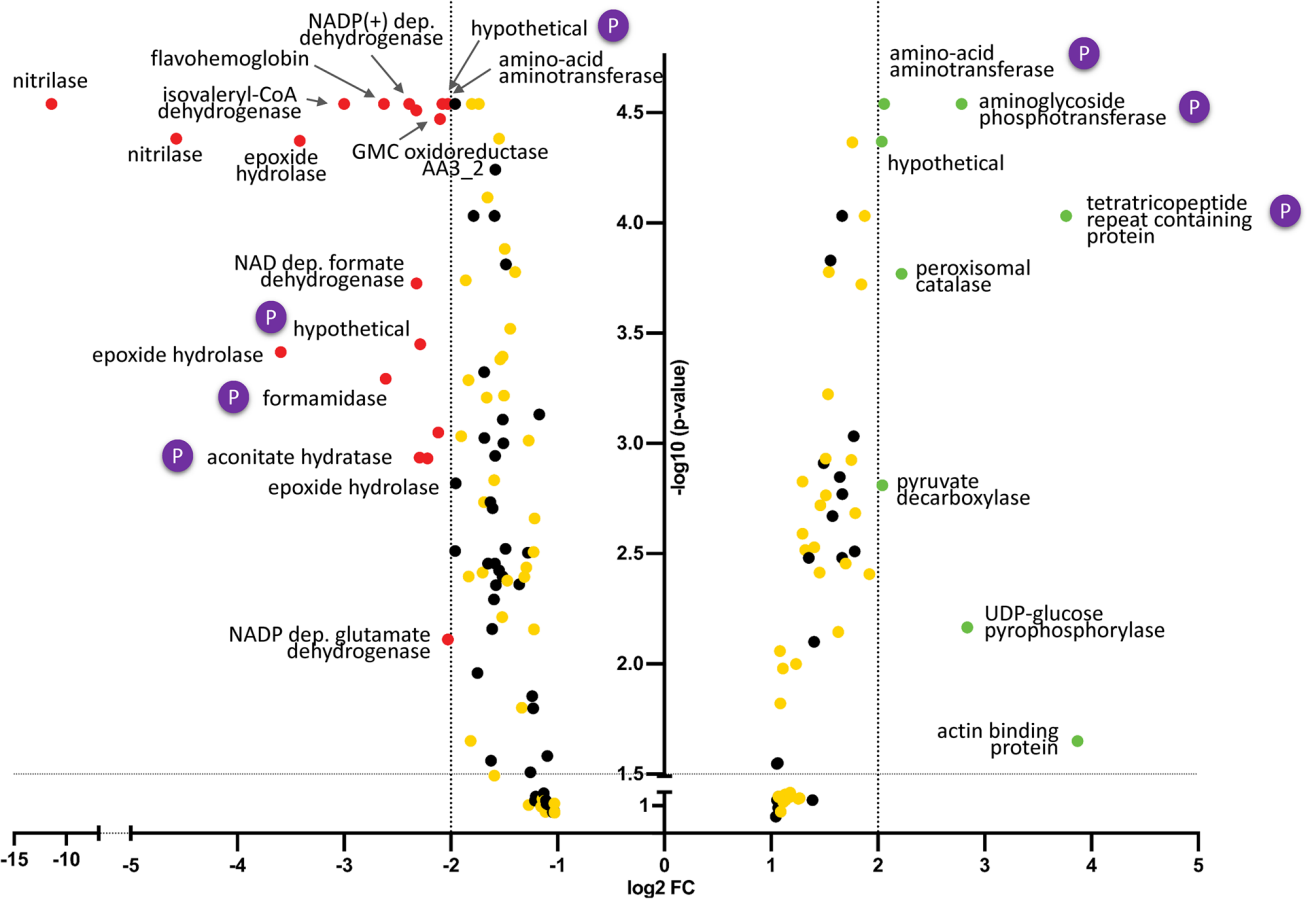
Volcano plot of all differentially regulated proteins in *T*. *atroviride* ∆*tmk1*-response compared to the WT-response to the host fungus *R. solani*. Red points: differentially expressed proteins that were significantly downregulated (Log_2_FC <  − 2; p < 0.05). Green points: differentially expressed proteins that were significantly upregulated (Log_2_FC > 2; p < 0.05). Yellow and black points: identified and non-identified proteins/isoforms of low significance |Log_2_FC| ≥ 1; p > 0.05), respectively. Dashed lines illustrate significance levels for protein expression of Log_2_FC and p-values. P indicates targets phosphorylated in at least one of the experiments.

Difference gel electrophoresis (DIGE) of the WT and mutant proteomes either challenged with *R. solani* or grown in self-confrontation resulted in the reproducible separation and detection of > 1200 protein spots. The proteomes of the ∆*tmk1* mutant and the WT showed different responses during self-confrontation. In total 70 protein spots were observed to be differentially regulated in the WT (28 upregulated, 42 downregulated) and 63 in the ∆*tmk1* mutant (39 upregulated, 24 downregulated), representing in total 119 individual gel spots (upregulated 69 individual spots, down regulated 50 individual spots). For all biologically relevant confrontation assays, i.e. WT and mutant in self-confrontation and in response to the host, we found in total 200 and 244 gel spots with up-regulation and down-regulation, respectively. From these 444 protein spots, 204 were identified by MS. From these 204 identified spots, a total of 159 were differentially expressed. These gel spots represent 115 individual proteins with at least one protein variant showing a |Log_2_FC| ≥ 1.5 (60 upregulated, 99 downregulated). Figure 2 shows differentially expressed proteins identified by mass spectrometry after DIGE separation and Table S4 summarizes expression level changes and protein identifications.

Within the protein spots that could be identified the mutant response to the host in comparison with the WT was rather moderate for most of the proteins |Log_2_FC| ≤ 3.3, except for HEX1, a hypothecial protein (Triat2_302977, sequence similarity to NADH dehydrogenase flavoprotein 2) and a NAD dependent formate dehydrogenase. HEX1 is unique to filamentous fungi saving it from cytoplasmic leakage^24^ and shows significantly stronger down-regulation in the mutant than in the WT. The strongly upregulated formate dehydrogenase is vital in the catabolism of C1 compounds. The WT showed only significant up-regulation for a putative actin binding protein.

The top 25 regulated proteins showed |Log_2_FC| between 11.4 and 1.9 (Table 2 provides information on the top 25 regulated proteins, i.e. |Log_2_FC| ≥ 2 for at least one of the proteoforms). The protein with the strongest differential regulation that was identified by mass spectrometry was a nitrilase. This protein was detected in more than one downregulated isoforms, being by this a good example for the increased complexity of the proteomic data. Post-translational modifications (PTMs), e.g. phosphorylation or acylation and acetylation, or PTMs in combination with amino acid point mutations are contributing to a shift in the isoelectric point (pI). However, no phosphopeptide was detected for nitrilase. Similar observations were made for NAD-dependent formate hydrognase, a putative aconitate hydratase, a hypothetical protein with sequence similarity to 3-octaprenyl-4-hydroxybenzoate carboxylyase, a branched-chain amino acid aminotransferase and NADP-dependent glutamate dehydrogenase (No. 13–15 and 23–24 in Table 2). All respective proteoforms showed the same overall expression (Log_2_FC between − 1.08 and − 2.32) (Details on regulation can be found in Table S4).

**Table 2:** List of the top 25 most robustly regulated candidate proteins in WT-response *vs* ∆*tmk1*-response (at least one isoform has a |Log_2_FC| > 2).

No.	Protein ID	Log_2_FC	phosphorylation	Annotation	FunCat number (second rank)	FunCat description
1	154783	− 11.42		Nitrilase	01.02// 32.05//32.07// 36.20	METABOLISM//nitrogen, sulfur and selenium metabolism// CELL RESCUE, DEFENSE AND VIRULENCE//disease, virulence and defense//detoxification// SYSTEMIC INTERACTION WITH THE ENVIRONMENT//plant / fungal specific systemic sensing and response
		− 4.57				
		− 1.79*				
2	162609	3.87		Putative actin binding protein	14.10// 16.01//16.17// 18.02// 20.09// 30.01//30.05//40.01//42.04// 43.01	PROTEIN FATE (folding, modification, destination)//assembly of protein complexes// PROTEIN WITH BINDING FUNCTION OR COFACTOR REQUIREMENT//protein binding//metal binding// REGULATION OF METABOLISM AND PROTEIN FUNCTION//regulation of protein activity// CELLULAR TRANSPORT, TRANSPORT FACILITIES AND TRANSPORT ROUTES//transport routes// CELLULAR COMMUNICATION/SIGNAL TRANSDUCTION MECHANISM//cellular signaling//transmembrane signal transduction//CELL FATE//cell growth / morphogenesis// BIOGENESIS OF CELLULAR COMPONENTS//cytoskeleton/structural proteins//CELL TYPE DIFFERENTIATION//fungal/microorganismic cell type differentiation
3	210492	3.76	x	Protein with tetratricopeptide repeat-containing domain	10.03//12.04// 42.16	CELL CYCLE AND DNA PROCESSING//cell cycle//PROTEIN SYNTHESIS//translation// BIOGENESIS OF CELLULAR COMPONENTS//mitochondrion
4	298020	− 3.59		Microtubule-binding protein	12.01//16.01//40.10//	PROTEIN SYNTHESIS//ribosome biogenesis//PROTEIN WITH BINDING FUNCTION OR COFACTOR REQUIREMENT (structural or catalytic)//protein binding//CELL FATE//cell death
		1.06				
		1.22				
5	131489	− 3.41		Putative epoxide hydrolase	01.05//32.07//32.10	METABOLISM//C-compound and carbohydrate metabolism//CELL RESCUE, DEFENSE AND VIRULENCE//detoxification//degradation / modification of foreign (exogenous) compounds
6	300623	− 3.00		Candidate isovaleryl-CoA dehydrogenase	01.01//01.06//02.11//02.25	METABOLISM//amino acid metabolism//lipid, fatty acid and isoprenoid metabolism//ENERGY//electron transport and membrane-associated energy conservation//oxidation of fatty acids
7	298303	2.84		Putative UDP-glucose pyrophosphorylase	01.03//01.04//01.05//02.01//02.19// 14.07	METABOLISM//nucleotide/nucleoside/nucleobase metabolism//phosphate metabolism//C-compound and carbohydrate metabolism//ENERGY//glycolysis and gluconeogenesis//metabolism of energy reserves (e.g. glycogen, trehalose)//PROTEIN FATE (folding, modification, destination)//protein modification
		− 1.12				
8	142072	2.78	x	Protein with protein-kinase like domain	99	UNCLASSIFIED PROTEINS
9	298717	− 2.63		Putative flavohemoprotein	01.02//02.13//20.01//32.01//32.05	METABOLISM//nitrogen, sulfur and selenium metabolism//ENERGY//respiration//CELLULAR TRANSPORT, TRANSPORT FACILITIES AND TRANSPORT ROUTES//transported compounds (substrates)//CELL RESCUE, DEFENSE AND VIRULENCE//stress response//disease, virulence and defense
10	299691	− 2.61	x	Formamidase-like protein	01.02	METABOLISM//nitrogen, sulfur and selenium metabolism
11	302658	− 2.39*		Putative NADP( +)-dependent dehydrogenase	01.05//01.06// 16.21	METABOLISM//C-compound and carbohydrate metabolism//lipid, fatty acid and isoprenoid metabolism// PROTEIN WITH BINDING FUNCTION OR COFACTOR REQUIREMENT//complex cofactor/co-substrate/vitamin binding
12	137374	− 2.33		Related to phospholipase A-2-activating protein	01.06// 10.01//10.03// 11.02// 14.13// 16.01// 18.02	METABOLISM//lipid, fatty acid and isoprenoid metabolism// CELL CYCLE AND DNA PROCESSING//DNA processing//cell cycle// TRANSCRIPTION//RNA synthesis PROTEIN FATE (folding, modification, destination)//protein/peptide degradation// PROTEIN WITH BINDING FUNCTION OR COFACTOR REQUIREMENT//protein binding// REGULATION OF METABOLISM AND PROTEIN FUNCTION//regulation of protein activity
13	302156	− 2.32		NAD dependent formate dehydrogenase	01.01//01.05//01.07//02.16	METABOLISM//amino acid metabolism//C-compound and carbohydrate metabolism//metabolism of vitamins, cofactors, and prosthetic groups// ENERGY//fermentation
		− 1.69				
		− 1.63				
		− 1.51				
14	150014	− 2.29	x	Putative aconitate hydratase	01.01//01.05// 02.10//02.16// 16.21//	METABOLISM//amino acid metabolism//C-compound and carbohydrate metabolism// ENERGY//tricarboxylic-acid pathway (citrate cycle, Krebs cycle, TCA cycle)//fermentation// PROTEIN WITH BINDING FUNCTION OR COFACTOR REQUIREMENT//complex cofactor/cosubstrate/vitamine binding
		− 1.55				
		− 1.24				
15	299540	− 2.29	x	Hypothetical protein	01.01//01.20	METABOLISM//amino acid metabolism//secondary metabolism
		− 2.08				
		− 1.80				
16	297668	2.22		Peroxisomal catalase	16.21// 20.01// 32.01//32.07	PROTEIN WITH BINDING FUNCTION OR COFACTOR REQUIREMENT//complex cofactor/cosubstrate/vitamine binding//CELLULAR TRANSPORT, TRANSPORT FACILITIES AND TRANSPORT ROUTES//transported compounds (substrates)// CELL RESCUE, DEFENSE AND VIRULENCE//stress response//detoxification
17	131489	− 2.22		Putative epoxide hydrolase	01.05// 32.07//32.10	METABOLISM//C-compound and carbohydrate metabolism// CELL RESCUE, DEFENSE AND VIRULENCE//detoxification//degradation / modification of foreign (exogenous) compounds
18	268974	− 2.12		Putative short-chain dehydrogenase/ reductase	01.05//01.06//01.20//16.21	METABOLISM//C-compound and carbohydrate metabolism//lipid, fatty acid and isoprenoid metabolism//secondary metabolism// PROTEIN WITH BINDING FUNCTION OR COFACTOR REQUIREMENT//complex cofactor/cosubstrate/vitamine binding//
		1.51				
		1.39*				
		1.27				
19	302655	− 2.10		GMC oxidoreductase AA3_2	01.06//01.07// 11.04// 14.13// 16.01//16.03// 34.11//	METABOLISM//lipid, fatty acid and isoprenoid metabolism//metabolism of vitamins, cofactors, and prosthetic groups//TRANSCRIPTION//RNA processing// PROTEIN FATE (folding, modification, destination)//protein/peptide degradation// PROTEIN WITH BINDING FUNCTION OR COFACTOR REQUIREMENT//protein binding//nucleic acid binding// INTERACTION WITH THE ENVIRONMENT//cellular sensing and response to external stimulus
20	302177	2.06	x	Branched-chain-amino-acid aminotransferase	01.01// 16.21//	METABOLISM//amino acid metabolism PROTEIN WITH BINDING FUNCTION OR COFACTOR REQUIREMENT (structural or catalytic)//complex cofactor/cosubstrate/vitamine binding
21	150078	2.04		Putative pyruvate decarboxylase	01.01//01.05//01.07//02.13//02.16// 11.02	METABOLISM//amino acid metabolism//C-compound and carbohydrate metabolism//metabolism of vitamins, cofactors, and prosthetic groups// ENERGY//respiration//fermentation// TRANSCRIPTION//RNA synthesis
22	297147	2.04		Putative Rho GDP-dissociation inhibitor	16.01//18.01//18.01.07//18.02//30.01// 34.07//40.01// 42.04// 43.01	PROTEIN WITH BINDING FUNCTION OR COFACTOR REQUIREMENT//protein binding//REGULATION OF METABOLISM AND PROTEIN FUNCTION//regulation by//CELLULAR COMMUNICATION/SIGNAL TRANSDUCTION MECHANISM//cellular signaling//INTERACTION WITH THE ENVIRONMENT//cell adhesion// CELL FATE//cell growth / morphogenesis// BIOGENESIS OF CELLULAR COMPONENTS//cytoskeleton/structural proteins// CELL TYPE DIFFERENTIATION//fungal/microorganismic cell type differentiation
23	146044	− 2.03*		Putative aminotransferase	01.01// 16.21	METABOLISM//amino acid metabolism// PROTEIN WITH BINDING FUNCTION OR COFACTOR REQUIREMENT//complex cofactor/cosubstrate/vitamine binding
		− 1.96				
		− 1.09*				
24	297025	− 2.03		NADP-dependent glutamate dehydrogenase	01.01//01.02// 02.10// 16.21// 42.01	METABOLISM//amino acid metabolism//nitrogen, sulfur and selenium metabolism// ENERGY//tricarboxylic-acid pathway (citrate cycle, Krebs cycle, TCA cycle)// PROTEIN WITH BINDING FUNCTION OR COFACTOR REQUIREMENT//complex cofactor/cosubstrate/vitamine binding//BIOGENESIS OF CELLULAR COMPONENTS//cell wall
		− 1.60*				
25	52055	− 1.96		Putative isocitrate dehydrogenase	01.01//01.05//01.06//01.07// 02.10//02.45// 16.21	METABOLISM//amino acid metabolism//C-compound and carbohydrate metabolism//lipid, fatty acid and isoprenoid metabolism//metabolism of vitamins, cofactors, and prosthetic groups//ENERGY//tricarboxylic-acid pathway (citrate cycle, Krebs cycle, TCA cycle)//energy conversion and regeneration// PROTEIN WITH BINDING FUNCTION OR COFACTOR REQUIREMENT//complex cofactor/cosubstrate/vitamine binding

Asterisk indicates proteins tentatively identified (statistical significance of search results for MS and MS/MS spectra not given, comparison of individual MS/MS spectra strongly indicate correct identification), Log_2_FC changes for protein isoforms are listed with the respective protein ID.

More than threefold up-regulation in the WT was observed for a putative actin-binding protein. Interestingly this protein was not differentially expressed in the ∆*tmk1* mutant. BLAST search revealed high sequence similarity to actin-binding cofilin from *T. guizhouense*. Actin-binding cofilin has been described to sever and depolymerize microfilaments in yeast^25^. The tetratricopeptide repeat-containing domain (sequence similarity to TIF31 of *T. gamsii*) is stronger, and more interestingly downregulated in the WT compared to the mutant (for details of Log_2_FC changes see Table S4) resulting in ∆*tmk1* response of Log_2_FC of 3.76. A microtubule-binding protein was detected as significantly upregulated for one isoform and slightly downregulated for two other isoforms. Microtubules are described to play significant transport roles in filamentous fungi^26^. The same applies for a 2.8-fold upregulated and 1.1-fold downregulated putative UDP-glucose pyrophosphorylase (UGPase). UGPase supports the conversion of UTP to UDP and shows stronger upregulation in the WT than the mutant. This observation correlates to findings for other strongly regulated proteins involved in metabolism, like the flavohemoprotein, a formidase-like protein, a putative NADP(+)-dependent dehydrogenase, and a protein related to phospholipase A2 activation (No. 9–12 in Table 2). A putative epoxide hydrolase and an isovaleryl-CoA-dehydrogenase candidate were downregulated. A branched chain amino acid aminotransferase was identified in multiple gel spots. GMC oxidoreducatase (No. 19), a pyruvate decarboxylase, and an isocitrate dehydrogenase (No. 25) as well showed moderate regulation in the mutant-host response, by this having an overall Log_2_FC of − 1.96 and − 2.10, respectively.

**Figure 3:**
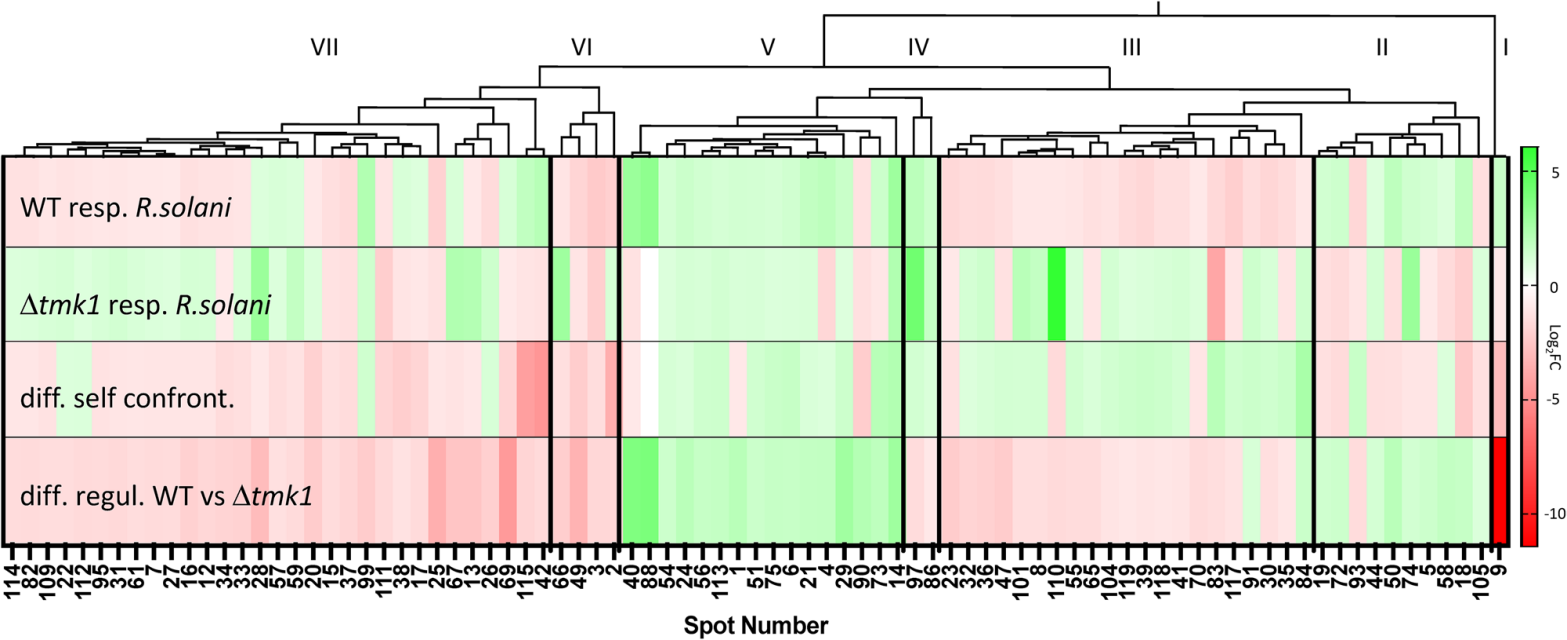
Grouped heatmap of 85 proteins (|Log_2_FC| > 1.5) showing different abundance in the WT and the ∆tmk1 mutant in self-confrontation or in confrontation with the host *R. solani*: ∆*tmk1-*response; WT-response; WT vs. ∆*tmk1* in self-confrontations; WT-response *vs* ∆*tmk1-*response to the host. (For protein identification, correlate protein spot number with identification listed in Table S4). The heat map was generated using GraphPad Prism version 10, GraphPad Software, Boston, Massachusetts USA, www.graphpad.com.

Figure 3 shows the grouped heat map generated by hierarchical clustering of these 85 identified proteins (protein names correlating with gel spots can be looked up in Table S4). It is obvious that nitrilase has an extremely reduced abundance in the ∆*tmk1* mutant upon confrontation with the host fungus (cluster I). Cluster II is defined by 10 proteins showing higher abundance in the WT but lower host-triggered abundance in the mutant upon interaction with *R. solani*. This cluster includes heat shock proteins of the HSP70 and HSP90 families, a ribosomal protein, a pyruvate and a phosphoglycerate kinase, a decarboxylase, a dehydrogenases, an oxidoreductase and a highly conserved translationally-controlled tumor protein (TCTP) orthologue. In *A. nidulans*, the TCTP TcpA has a role in hyphal branch establishment during vegetative growth and in balancing asexual and sexual differentiation^27^. 21 proteins fall into the cluster III exhibiting reduced abundance at host contact compared to the self-confrontation control in the WT but increased abundance in the ∆*tmk1* mutant. These include 13 metabolic proteins such as enzymes involved in glycolysis, the citric acid cycle, or proteins relevant for electron transport, HSP binding and the transfer of sulfur-containing groups. This cluster also contains HEX1 protein (see above). Cluster IV is formed by a pyruvate kinase and the hypothetical protein with sequence similarity to NADH dehydrogenase flavoprotein 2 by showing up regulation for both the mutant and the WT in response to *R. solani*. These proteins are involved in metabolism and energy conservation. Cluster V consists of 16 proteins with lower abundance in the ∆*tmk1* mutant compared to the WT whose expression is upregulated by the presence of the fungal host only in the WT but not the mutant. While this cluster mainly contains proteins distributed in diverse functional categories, two proteins represent members of the GDP dissociation inhibitor family, three are involved in metabolic processes, three in GTP binding, GTP biosynthesis or have GTPase activity. Three out of four members of cluster VI are candidates involved in the C-compound and carbohydrate metabolism showing up-regulation only for the ∆*tmk1* mutant. With 31 members cluster VII is the largest cluster identified. It, amongst others, comprises 21 proteins involved in metabolism, mainly members of the citric acid cycle, metabolism of amino acids and C-compounds/carbohydrates, and seven proteins with a putative role in cell rescue, defence and virulence. Interestingly, a protein variant of TCTP (increased molecular weight) was also identified showing up-regulation in the mutant. In addition, five proteins involved in electron transport are contained in this cluster, two of which were isoforms of a pyroverdine/dityrosine biosynthesis protein involved in fungal cell type differentiation.

### The phosphoproteome of *T. atroviride*

The ∆*tmk1* mutant showed reduced mycoparasitic activity against host fungi^18^ suggesting that phosphorylation of target proteins acting downstream of Tmk1 is involved in full activation of the mycoparasitic response in *T. atroviride*. Three approaches were used to study the phoshoproteome of *T. atroviride* upon host contact. Fluorescence staining of phosphoproteins after DIGE separation resulted in the detection of 141 spots with differential fluorescence between the WT and the ∆*tmk1* mutant during the fungal host interaction; of these, 19 proteins were identified by mass spectrometry. The shotgun phosphoproteomics approach after TiO_2_-enrichment resulted in additional 9 phosphoproteins from the confrontations of *T. atroviride* WT and the ∆*tmk1* mutant against *R. solani*. The third approach, based on SDS-PAGE and TiO_2_-enrichment of proteins extracted from all tested conditions revealed 10 phosphoproteins. In total, 33 phosphoproteins could be identified from *T. atroviride* which are associated with cellular metabolism and energy production, protein synthesis and fate, and organization of actin filaments (details see Table S4) and which constitute the first partial phosphoproteome reported for the mycoparasitic interaction of *Trichoderma*. We identified a protein containing the tetratricopeptide repeat domain and the branched-chain-amino-acid aminotransferase to be phosphorylated. Proteins with tetratricopeptide-like helical domains are involved in a variety of biologically relevant processes including cell cycle regulation, transcriptional control, mitochorndrial and peroxisomal protein transport, neurogenesis and protein folding. The latter was described to control the conserved target of rapamycin complex 1 signalling in *S. cerevisiae*^28^*.* Two other downregulated phosphoproteins belong to amino acid metabolism as well, an aconitate hydratase (two spots in DIGE differing by ∆pI 0.74 indicate the addition of one phosphorylation) and a hypothetical protein with an amino acid sequence similarity to 3-octaprenyl-4-hydroxybenzoate carboxy-lyase from *T. guizhouense* (three spots in DIGE differing by ∆pI 0.21 and 0.28). Both of them are among the top 25 regulated proteins identified in this study (Table 2). The downregulated formamidase-like protein is part of the nitrogen, sulfur and selenium metabolism, one upregulated candidate is associated with cell division, and one upregulated proteoform was assigned as a putative aminoglycoside phosphotransferase. Comparative SDS-PAGE analysis resulted in 69 different proteins, but only 12 were found in all technical replicates. From these 69 proteins, 13 could be identified and six were also found in the DIGE approach, showing down regulation for C-compound and carbohydrate metabolism (three proteins), protein synthesis (one protein) and amino acid metabolism (one protein). For all proteins the phosphorylated form of the peptide was detected. The shotgun analysis gave the least informative results. However, the presence of translation elongation factor 3 (detected in comparative SDS-PAGE and DIGE, upregulated, protein synthesis) and a phosphoglucomutase (detected in DIGE, one spot up- and one downregulated in DIGE, C-compound and carbohydrate metabolism) in their phosphorylated form was corroborated.

## Discussion

### General expression response of *T. atroviride* to the presence of *R. solani*

For both transcriptomics and proteomics profiling, the *T. atroviride* WT strain and the ∆*tmk1* mutant were cultivated in direct confrontation assays with themselves (control) and with the living host fungus *R. solani* (induced sample) and the *Trichoderma* mycelia were harvested upon first contact between the two fungi. 62% of genes strongly regulated in *T. atroviride* WT upon early contact with *R. solani* were found to be upregulated (88/142). The genes with the strongest regulation comprised several candidates encoding enzymes involved in cellular redox reactions, including oxidoreductases (FAD-dependent monooxygenase, peroxidases, ketol-acid reductoisomerase), putative short-chain dehydrogenases and alcohol dehydrogenases.

For being able to attack and parasitize its host, *Trichoderma* has to attach to host hyphae accompanied by the production of cell wall degrading enzymes for host lysis. Among the strongly regulated genes during the contact of *T. atroviride* and *R. solani,* several candidates with a putative function in host fungus attack were identified. The high differential expression of genes containing carbohydrate-binding WSC domains and genes coding for SSCPs suggests their potential roles in mycoparasitism. So far, elicitor-like SSCPs such as *T. virens* Sm1 (EPL1 in *T. atroviride*) have been shown to induce systemic disease resistance in plants, whereas for ectomycorrhizal basidiomycetes SSCPs appear to be rather important in symbiotic interactions; reviewed in Druzhinina et al*.*^29^.

There are several reports on the host-induced expression of chitinases and proteases in *T. atroviride* during mycoparasitism^15,17,18,30^. We found these enzymes, putatively involved in host lysis, to be downregulated during the early response of *T. atroviride* to *R. solani*, including a putative secreted phospholipase, a chitosanase, a chitinase with a CFEM domain, and chitinase CHI 18-2.

The ability of the mycoparasite to defend itself against substances derived from the fungal host is reflected by the regulation of several genes involved in stress response and detoxification. In *T*. *atroviride* confronted with *R. solani* we detected strong upregulation of a FAD-monooxygenase, which specializes in the oxidation of xeno-substrates in order to facilitate the excretion of these compounds from living organisms. Several stress response and detoxification genes were strongly downregulated, such as RTA1-like proteins involved in resistance to toxic substances, glutathione-dependent formaldehyde-activating enzyme, a phenylacrylic acid decarboxylase-like protein, a HSP20 family protein employed in stress response, a thioredoxin-like protein important in defense against oxidative stress, and a protein similar to CipC antibiotic response protein.

Among the individual candidates strongly upregulated during the early *T. atroviride* response to the host fungus were two genes encoding elongation factors (EF1 and EF2; Table S1), several genes implicated in cellular transport processes (e.g. MFS transporters, a monosaccharide transporter, Golgi vesicular transport protein, vacuolar sorting proteins), and in primary carbon metabolism and energy production (e.g. pyruvate kinase, fumarate hydratase, succinate dehydrogenase). Importantly, components of MAPK signaling pathways were among the genes strongly upregulated during the response of *T. atroviride* to *R. solani*. Besides a GprK-type GPCR, a class XIII and a PTH11-type receptor, Tmk2 and a component of the osmosensing pathway, the Pbs2 MAPKK, showed elevated transcription upon contact with the host. Accordingly, MAPKs have previously been found to be involved in the antagonistic action of different *Trichoderma* species^18,31–35^.

### The Tmk1 MAP kinase governs the expression of mycoparasitism-related genes and proteins as well as components of other signaling pathways

In this study, the differential proteome between the WT and the ∆*tmk1* mutant was analyzed by 2-dimensional difference gel electrophoresis (DIGE) which resulted in the identification of 115 proteins by mass spectrometry, presented in 158 individual gel spots. 88 protein isoforms showed a differential abundance of ≥ 1.5-fold-change in at least one of the tested conditions. Most of these differentially regulated proteins are supposed to be involved in processes such as Metabolism and Energy. Three enzymes associated with the tricarboxylic acid (TCA) cycle showed reduced abundance in *T. atroviride* WT but not the ∆*tmk1* mutant during confrontation with the host fungus compared to the self-confrontation control, whereas pyruvate decarboxylase showed higher abundance under mycoparasitism conditions in the WT but was downregulated in the mutant. Pyruvate decarboxylase catalyses the conversion of pyruvate to acetaldehyde and its activity is closely associated with ethanol production in *Aspergilus nidulans* and with lipid accumulation in aerial mycelia of *Giberella zeae*^36^. The finding that *T. atroviride* WT reorients its carbon catabolism towards the PAA (pyruvate-acetaldehyde-acetate) and fermentation pathways in a host-triggered fashion differs from the behaviour of the ∆*tmk1* mutant which showed unaltered expression of TCA cycle enzymes irrespective of contact with the fungal host.

The protein with the strongest differential abundance between the WT and the ∆*tmk1* mutant was identified as cyanide hydratase/nitrilase. The highly reduced expression of this protein upon host contact in the mutant was also reflected at the transcriptome level (WT-response *vs* ∆*tmk1-*response: down-regulation with Log_2_FC − 5.4). Nitrilases catalyse the hydrolysis of nitrile (R-CN) compounds to the corresponding carboxylic acid and ammonia and play critical roles in plant–microbe interactions for defence, detoxification, nitrogen utilization, and plant hormone synthesis^37^. In one of our previous studies this nitrilase was, however, one of the most strongly induced genes in *T. reesei* during the sensing and overgrowth of *R. solani* and the second most upregulated gene in *T. virens*^15^. The fungal cyanide hydratases form a functionally specialized subset of the nitrilases which catalyse the hydrolysis of cyanide to formamide^38^ and could be involved in the defence against cyanide produced by other soil microorganisms such as fungi and bacteria^39^.

As the role of the Tmk1 MAPK in *T. atroviride* mycoparasitism indicates that protein phosphorylation is involved in the cellular activities during host recognition and attack, we aimed to study the phosphoproteome of the fungus. To identify potentially regulated phosphoproteins, DIGE analysis was supplemented by a phosphoprotein gel stain after DIGE separation, identification from the total protein extract after phosphopeptide enrichment via TiO_2_ (shotgun phosphoproteomics), and identification of phosphopeptides after SDS-PAGE separation. 33 proteins were identified based on peptide analysis and classified either to cellular metabolism, energy production, protein synthesis and fate, or cell organization. Two FAD-binding-domain containing proteins, a translation elongation factor 3-like protein, a transaldolase involved in carbohydrate transport and metabolism, a heat shock protein 70 (Hsp70), an actin, a protein similar to GDP dissociation inhibitor Gdi1, that regulates vesicle traffic in secretory pathways of *Saccharomyces cerevisiae*^40^, and a chorismate synthase were phosphorylated and were detected also in the DIGE experiments. Hsp70s are an important part of the cell’s machinery for protein folding, performing chaperoning functions, and helping to protect cells from the adverse effects of physiological stress^41^. A recent study^42^ reported on the time-dependent global phosphoproteome change in *T. reseei* following carbon source exchange. The study also showed a significant number of phosphoproteins to be involved in amino acid transport and metabolism and proteins related to carbon storage showed a significant increase in phosphorylation. Our data on mycoparasitism-induced phosphoproteome regulation give first insights into phosphorylation events occurring at the early contact of *T. atroviride* with *R. solani,* paving the way to a better understanding of Tmk1-mediated signalling on the protein level.

The Tmk1-dependent transcriptional response upon early host contact identified 140 genes strongly affected by Tmk1 during mycoparasitism. A very large proportion (89%) of these genes indicated a mainly stimulating function of the Tmk1 MAPK in *T. atroviride* during early mycoparasitism. We detected genes coding for a fumarate hydratase, an FAD-dependent oxidoreductase, an elongation factor 2, a GT glycosyltransferase, a cross-pathway control protein CPC1/Gcn4, and a serine palmitoyltransferase among the top ten most robustly identified candidates (Table 1). Among the genes for which Tmk1 most robustly repressed a mycoparsitism response were isochorismatase hydrolase, a putative NADH dehydrogenase, a glyoxalase-like family protein, a WSC domain-containing protein, and the RTA1 like protein, with strong downregulation only in the WT response. These were complemented by the glucose-repressible protein Grg1 and three hypothetical proteins, with strong upregulation only in the mutant response. Notably, *grg-1* is one of the earliest expressed genes in the *N. crassa* conidiation program with its expression being regulated by extracellular glucose levels, circadian rhythm, and blue light^43^. The gene with the highest downregulation in the WT response of *T. atroviride* to *R. solani* encoded an isochorismatase family protein; however, no regulation of this gene in response to the host was detected in the ∆*tmk1* mutant. We speculate that such genes (in our study furthermore those encoding FAD-monooxygenase, zinc-containing alcohol dehydrogenase superfamily protein, putative NADH dehydrogenase and a PTH11-type GPCR receptor; all UP.DownWT.RbtMut; Table S2) are strongly dependent on Tmk1 and lack regulation when Tmk1 is missing regardless of environmental signals. Isochorismatases catalyse the conversion of isochorismate to 2,3-dihydroxybenzoate and pyruvate, and are involved in the synthesis of phenazine by *Pseudomonas aeruginosa*^44^ and the siderophore enterobactin by *Escherichia coli*^45^. Proteins with an isochorismatase motif are present in all filamentous ascomycetes but have been found to be secreted mainly in phytopathogens^19^. Together with the fact that isochorismate is a precursor of salicyclic acid, a metabolite mediating plant defense, this raised the speculation that isochorismatases secreted by fungi could act to reduce salicylic acid accumulation in response to pathogen attack and thus inhibit plant defense responses. Zhu et al*.*^22^ showed that *V. dahliae* isochorismatase hydrolase ICSH1 is a virulence factor that contributes to interference with potato’s salicylate and jasmonate defense signaling. The identified *T. atroviride* isochorismatase hydrolase is predicted to be a secreted enzyme; however, in contact with *R. solani* the corresponding gene was downregulated in the WT response. The fact that this candidate was not regulated in the ∆*tmk1-*response to the host fungus suggests that it is governed by the Tmk1 signaling pathway. It was the most suppressed secreted target identified in this study (Fig. 1).

In general, a stimulatory effect of Tmk1 during the early mycoparasitism response was observed at the transcriptional level (Fig. 1). The expression of genes coding for nitrilase NIT2, putative flavohemoprotein-like protein, 6-phosphogluconate dehydrogenase, putative ketol-acid reductoisomerase, actin, elongation factor 1 gamma and glutathione S-transferase domain-containing protein, aconitate hydratase, pyruvate kinase pykF, aldehyde dehydrogenase, metalloexopeptidase and bifunctional catalase/peroxidase were found to be significantly affected by Tmk1 in our transcriptome experiments, and the respective protein expression levels corroborated that observation. *Tmk1* deletion also affected the expression of genes involved in signaling pathways. With the exception of a PTH11-type fungal GPCR, all other identified signaling-related candidates such as a GprK-type GPCR, the Tmk2 MAP kinase, the Pbs2 MAP kinase kinase, a putative member of the 14-3-3 protein family, a Las1-domain-containing protein, and a putative beta-tubulin, their responses to mycoparasitism were suppressed by Tmk1. The MAPKK Pbs2 was strongly upregulated in the WT, but downregulated in the mutant. Pbs2 is involved in a signal transduction pathway that is activated by changes in the osmolarity of the extracellular environment and phosphorylates Hog1 on a tyrosine residue^20^. Tmk2 (strongly upregulated in the WT, but downregulated in the mutant) is an orthologue of the yeast cell wall integrity pathway MAPK Slt2p^5^. Similar to yeast, this MAPK governs cell wall integrity in *T. reesei* and *T. virens*^33,46^; however, its function in *T. virens* might overlap with TmkA (Tmk1 homolog) as both deletion mutants showed a reduced ability to antagonize *Sclerotium rolfsii* while retaining the ability to fully overgrow and parasitize *R. solani*^33^. 14-3-3 proteins (of which in our study a candidate was strongly upregulated in the WT, but downregulated in the mutant) are structurally similar phospho-binding proteins that regulate essentially every major cellular function. They have the ability to bind a multitude of functionally diverse signaling proteins, including kinases, phosphatases, and transmembrane receptors. 14-3-3 proteins are found in all eukaryotic cells and are a critical part of signaling pathways that govern processes such as apoptosis, cell cycle progression, autophagy, glucose metabolism, and cell motility^47^. The 14–3-3 proteins BMH1 and BMH2 in *S. cerevisiae* are positive regulators of rapamycin-sensitive signaling via TOR kinases while they play an inhibitory role in Rtg3p-dependent transcription involved in retrograde signaling^48^.

**Figure 4:**
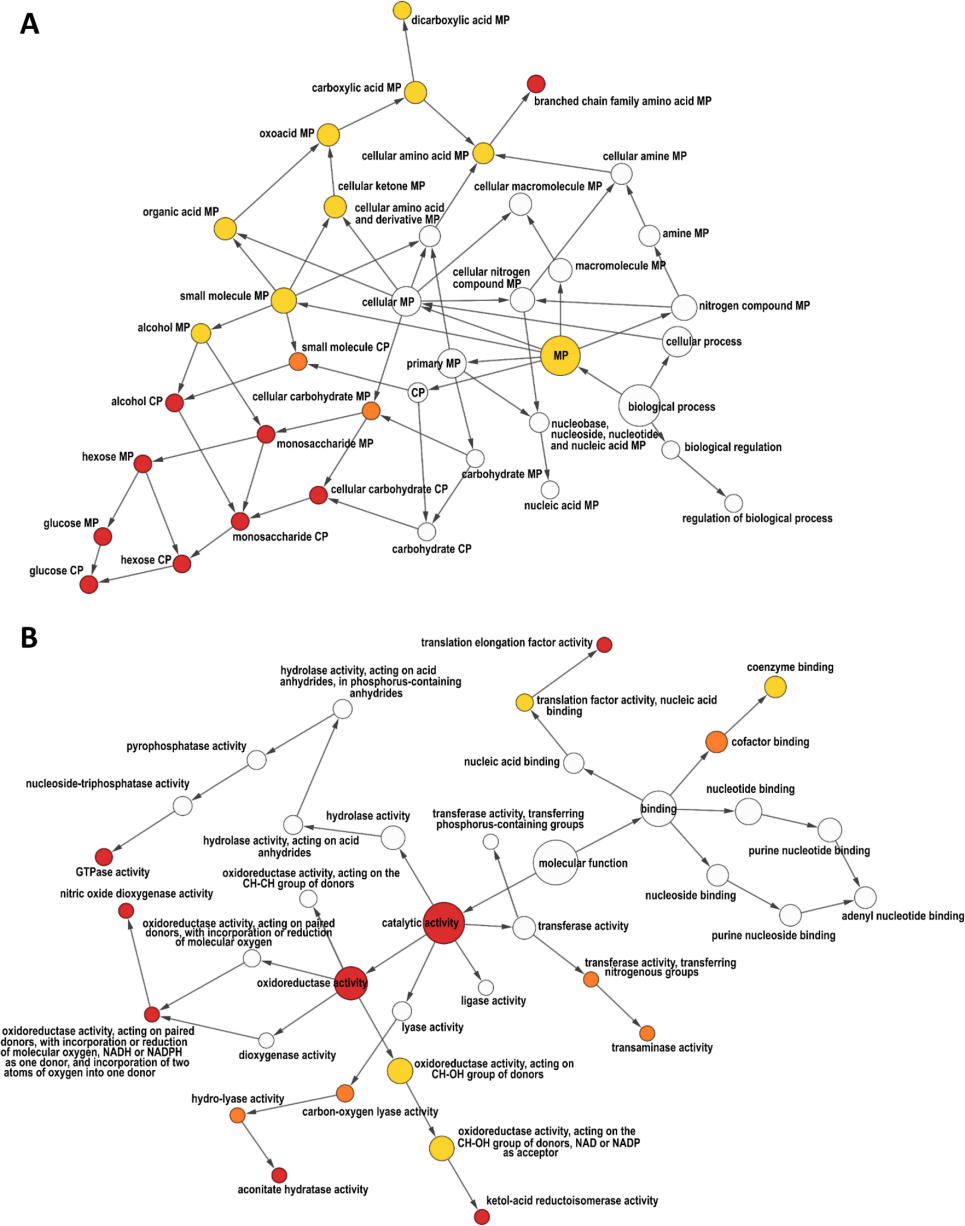
Proteome-transcriptome intersection networks of Gene Ontology (GO) enrichment analyses for biological process (**A**) and molecular function (**B**) categories in the ∆*tmk1* mutant during the confrontation with *R. solani* compared to the WT-response. The colored circles mark groups with significant overrepresentation of genes (*p*-value ≤ 0.05) with yellow, orange and red corresponding to significant enrichment in only proteome, only transcriptome and both datasets, respectively. The size of circles corresponds to the sum of cluster frequencies, listed in Table S5 MP and CP abbreviate metabolic and catabolic process respectively.

Intersection networks from our separate GO term enrichment analyses performed with BiNGO^49^ and visualized with Cytoscape59⁠ v3.7.2^50^ resulted in several groups of processes for which genes as well as proteins were found to be overrepresented on a global level. We revealed an involvement of Tmk1 in branched-chain amino acid metabolic processes, alcohol catabolic process, and glucose metabolic and catabolic process (Fig. 4A; *p*-value ≤ 0.05). Small molecule metabolic processes such as those of dicarboxylic acid, and in general the metabolic process as a main category, were significantly overrepresented only for targets detected in the differential proteome. At the level of molecular function, during the response to the host fungus Tmk1 governed targets with translation elongation factor activity that function in chain elongation during polypeptide synthesis at the ribosome as well as targets with general catalytic activity (Fig. 4B, Table S5). In addition, we found specific overrepresentation of Tmk1 targets with GTPase activity, aconitate hydratase activity and general oxidoreductase activity. Small GTPases typically function as nodal points that integrate broad upstream regulatory inputs and disseminate broad effector outputs^51^. Further, targets assigned to oxidoreductase activity acting on paired donors with incorporation or reduction of molecular oxygen, NADH or NADPH as one donor and incorporation of two atoms of oxygen into one donor; nitric oxide dioxygenase activity, and ketol-acid reductoisomerase activity (enzymes part of alcohol oxidoreductases) were significantly enriched in both datasets (Fig. 4B; *p*-value ≤ 0.05). The balance between reactive oxygen species and reactive nitrogen species production by the host and stress response by fungi is a key axis of the host–pathogen interaction^52^. Oxidoreductases catalyze the transfer of electrons from reductant (electron donor) to the oxidant (electron acceptor) and usually utilize NADP+ or NAD+ as cofactors. Part of this group are free radical and reactive oxygen detoxifying enzymes that include nitric oxide dioxygenase (EC 1.14.12.17), superoxide dismutase (EC 1.15.1.1), catalase, and peroxidase (EC 1.11.1). Nitric oxide (NO) is a signaling molecule that can be lethal to cells by poisoning cellular energy production and its most sensitive targets are aconitases, enzymes that catalyzes the isomerization of citrate to isocitrate in the citric acid cycle, and cytochrome oxidase, the last enzyme in the respiratory electron transport chain of mitochondria. We found these enzyme groups significantly enriched in the transcriptome and proteome datasets, indicating that Tmk1 may govern targets responsible for molecular functions that prevent reactive oxygen stress, particularly nitrosative stress, and might be directly or indirectly part of NO but also H_2_O_2_ signaling.

## Materials and methods

### Fungal strains and growth conditions

*T. atroviride* strain P1 (ATCC 74058) and the derived MAP kinase deletion mutant ∆*tmk1*^18^ were used in this study. The parental as well as the mutant strains were maintained on potato dextrose agar (PDA; (BD Dicfo, Franklin Lakes, NJ) and PDA supplemented with 200 µg/ml hygromycin B, respectively.

### Dual confrontation assays and RNA isolation

Plate confrontation assays using *Rhizoctonia solani* (pathogenic isolate obtained from the collection of the Department of Agricultural Sciences, Università degli Studi di Napoli “Federico II”, Naples, Italy) as host fungus were performed on PDA covered with a sterile cellophane membrane at 25 °C and a 12 h light–dark cycle as previously described^53^. Self-confrontations between the *Trichoderma* strains tested served as un-induced controls. The plates were incubated until the mycelia of both fungi made first contact and the *Trichoderma* mycelium was harvested from the confrontation zone (5 mm of the peripheral area) from 10 independent plates, which were considered as biological replicates. The universal reference sample for microarray analysis was obtained as previously described^16^. Mycelia were ground to a fine powder under liquid nitrogen and total RNA was extracted with the PeqGOLD TriFast DNA/RNA/Protein Purification Reagent (PEQLAB Biotechnology, VWR) followed by further purification using the RNeasy MiniElute Cleanup Kit (Qiagen, Valencia, CA, USA). RNA integrity was checked using an Agilent 2100 Bioanalyzer (Agilent, Santa Clara, CA, USA).

### Microarray design and data analysis

Gene expression profiling was performed with a custom high-density microarray platform for genome-wide transcriptional profiling of the 11,863 genes listed in the Gene Catalogue of version 2 of the *T. atroviride* genome database [http://genome.jgi.doe.gov/Triat2/Triat2.info.html] as described in Atanasova et al*.*^16^. Each confrontation sample was hybridized against a universal reference sample as described by Atanasova et al*.*^16^ in four replicates, and statistical analyses performed using R (www.r-project.org) and Bioconductor libraries (www.bioconductor.org). An empirical Bayes regularized *t*-test in the Bioconductor *limma* framework was applied for identifying differential expression after conservative Benjamini-Yekutieli correction for multiple testing for strong control of the false discovery rate (FDR) to *q* < 5%. Mycoparasitism-relevant Tmk1 target genes were identified by evaluation of the responses to the host fungus of the *T. atroviride* WT strain (WT-response = “WT + host fungus” *versus* “WT + WT”) as well as the ∆*tmk1* mutant (∆*tmk1*-response = “∆*tmk1* + host fungus” *versus “*∆*tmk1* + ∆*tmk1”*) by comparing gene expression levels of the respective strains upon growth under non-mycoparasitic (self-confrontation control) and mycoparasitic conditions. The ∆*tmk1*-response was then compared to the WT-response resulting in a gene set comprising genes being targeted by Tmk1 under mycoparasitic conditions (WT-response *vs* ∆*tmk1-*response). To allow statistical significance tests to combine evidence across samples, this comparison was directly computed as a specific contrast in the linear model. The analysis was performed for gene transcripts and for functional groups assigned via FunCat^54^. Rank-product meta-analysis was performed to combine evidence from a range of normalization approaches. On a second level (WT-response *vs* ∆*tmk1-*response), candidate genes with a large average effect strength, employing a conservative threshold of |aLog_2_FC|> 2 were considered like in Atanasova et al*.*^16^. The genes were afterwards separated in seven sub-groups of genes based on their first level responses (WT-response [WT] and ∆*tmk1-*response [Mut]). The genes with a negative difference in the second level contrast WT-response *vs* ∆*tmk1-*response fall into four groups: (1) genes strongly upregulated in the WT-response and strongly downregulated in the ∆*tmk1-*response (DOWN.UpWT.DownMut); (2) genes where both of the responses showed strong downregulation and the downregulation was much stronger in the mutant (DOWN.DownWT.DownMut); or genes where one of the strains was not regulated (response below the threshold |aLog_2_FC| > 1), Rbt): either (3) DOWN.RbtWT.DownMut or (4) DOWN.UpWT.RbtMut. The genes with a positive difference in the second level contrast conversely fall into four groups where genes were either 1) strongly downregulated in the WT and upregulated in the mutant (UP.DownWT.UpMut) or one of the strains was upregulated and the other was regulated below threshold (2: UP.DownWT.RbtMut or 3: UP.RbtWT.UpMut). Gene Ontology enrichment analysis of genes implicated in the transcriptome and proteome data sets were individually performed using BiNGO^49^. Multiple testing correction to search for significant differences in frequencies of their GO terms compared to all *T. atroviride* gene models (extracted from JGI Mycocosm^55^) was done using Benjamini and Hochberg False Discovery Rate (FDR ≤ 0.05) correction. Results of both datasets were visualized using Cytoscape59⁠ v3.7.2^50^ and merged intersection networks based on *p*-value ≤ 0.05 were extracted.

In addition to automatic gene annotation, every differentially expressed gene was manually curated and its protein domains were manually checked for Pfam and InterPro classification using NCBI protein blast (https://blast.ncbi.nlm.nih.gov/Blast.cgi) versus the NCBI non-redundant (NR) protein database (09/07/2020) and InterproScan sequence search (https://www.ebi.ac.uk/interpro/search/sequence-search). Transmembrane helices and signal peptide cleavage sites were predicted using CBS server tools TMHMM Server v. 2.0^56^ and SignalP 4.1^57^ employing default parameters. TargetP v. 2.0 server was used for prediction of subcellular location of proteins^58^.

### Protein extract preparation

50 mg of wet mycelia harvested from different individual plates was pooled, suspended in 0.8 mL buffer (50 mM Tris–HCl pH 8.0; 10% v/v glycerol; 0.5% v/v Tween20; 2 mM DTT; 10% Toluol and 0.5% HaltTM Protease and Phosphatase Inhibitor Cocktail) and lysed under sonication (Branson Ultrasonics, Brookfield, CT, US) at 4 °C. Cell debris was removed by centrifugation (14,000×*g*, 10 min, 4 °C) and proteins were precipitated with pre-cooled 4.71 µM trichloroacetic acid (TCA) and acetone (both − 20 °C) added to the sample at a volume ratio of 8/1/1. Protein precipitate was recovered by centrifugation after letting the solution sit at − 20 °C for 60 min. Pellets were washed with cold acetone and dried at ambient conditions for 30 min. After overnight re-solubilization with isoelectric focusing (IEF) buffer (7 M urea, 2 M thiourea, 4% CHAPS, 30 mM Tris–HCl pH 8.5 and 10 µL/mL HaltTM Protease and Phosphatase Inhibitor Cocktail) at 4 °C the pH was adjusted to 8.5. Bradford assay was used to determine the protein concentration of all samples and aliquots were stored at − 20 °C until further use.

### 1-Dimensional gel electrophoresis (SDS-PAGE)

SDS-PAGE was carried out using 8 × 7 × 0.1 cm ready-made gels (10% T, Tris–Glycine buffer) in a XCell*SureLock*® (all Invitrogen, Carlsbad, CA, US) according to the manufacturers instruction. 30 µg protein extract was mixed with Tris–Glycine sample buffer (Invitrogen) to reach a final buffer concentration of 62.5 mM Tris–HCl, 2% SDS, 10 vol% glycerol, 0.1 M DTT and 0.01% bromophenol blue, pH 6.8. The sample was reduced for 2 min at 96 °C before analysis.

### 2-Dimensional gel electrophoresis (2D GE) for western blot analysis and phosphoprotein staining

The total of 45 µg protein extract was mixed with 1 M DDT and IPG-buffer (pH 3–10 NL, GE Healthcare, Chicago, IL, US) to get final concentrations of 20 mM DTT; 2 vol% ampholytes were added. IEF buffer was added to reach a final volume of 125 µL. 7 cm IPG strips (NL 3–10) were rehydrated with the sample overnight in the dark. A step gradient was used for focusing until a total of 36 kVh was reached using a Multiphor II system (GE Healthcare). IPG strips were first reduced with 6.5 mM DDT and then alkylated with 132 mM iodacetamide (IAA) in equilibration solution (6 M urea, 75 mM Tris–HCl pH 8.8, 29.3% glycerol, 2% SDS and 0.002% bromophenol blue) for 15 min each. The second dimension was performed as described above (SDS-PAGE). Image analysis was carried out using DeCyder v7.0. For spot detection a maximum spot number was set to 2000 and the selection algorithm 6.0 was chosen. The exclusion filter was set for a slope of at least 2.5 and a spot volume above 50,000. Four technical replicates for each sample were used to determine average abundance differences and perform Student’s *t*-test. The gel with the highest spot count was assigned as master gel (Supp. Fig. S1).

### Phosphoprotein staining

The staining of the 2D gels was carried out according to the manufacturer’s instructions using the PhosDecor™ Fluorescent Phosphoprotein In-Gel Detection Kit (Sigma Aldrich, St. Louis, MO, US). Fluorescence images were acquired on a Typhoon FLA9000 (GE Healthcare) (resolution 100 µm, 532 nm, LPG-filter). Gel images were pre-processed with Image Quant v7.0 and image analysis was carried out using DeCyder v7.0 (GE Healthcare). Spots of interest were cut out and identified by MS (details see “In-gel Tryptic Digestion and protein identification” section).

### Western blot analysis

After SDS-PAGE, proteins were transferred on a membrane using a XCell*SureLock*® (Invitrogen) device. The gel was equilibrated for 30 min in the transfer buffer (0.2 M glycine, 0.025 M Tris, 0.001 M Na-EDTA, 0.002 M SDS and 20% v/v methanol). Nitrocellulose was used as blotting membrane (Trans-Blot® Transfer Medium; 0.45 µm). Blotting was carried out at constant current (200 mA) for 2 h. The detection of MAPK proteins on the 1D- and 2D-gels was performed with the PhosphoPlus® p44/42 MAPK (Erk1/2) (Thr202/Tyr204) Antibody Kit (ThermoFisher, Waltham, MA, US) following the manufacturer’s instructions.

### Difference gel electrophoresis (DIGE) approach

Cy2 and Cy3 (GE Healthcare) were used for labelling (100 fmol/80 µg protein), of which Cy2 was used for the internal standard (pooled samples) and Cy3 for the samples. After mixing equal amounts of sample and internal standard, 20 mM DTT, 2 vol% IPG-Buffer (pH 3–10 NL) and IEF buffer were added to a final volume of 450 µL. Samples were loaded via in-gel hydration onto 24 cm IPG strips (NL 3–10) overnight and subsequently focused until a total of 67 kVh was reached. Horizontal gel electrophoresis was performed in a HPE-Tower (Serva, Heidelberg, D) using ready-made gels (12.5%T, 25.5 × 20 cm, Serva) after equilibrating the IPG strips in the equilibration buffer containing 0.83% w/v DTT first and then in 2.1% w/v IAA. Four technical replicates were acquired, scanned, and processed as described above (FLA9000, Quant v7.0). Statistical analysis was carried out using DeCyder v7.0 (maximum spot number 2000, exclusion filter: slope ≥ 2.5, spot volume > 50,000, average abundance difference calculated from technical replicates). Protein statistics was calculated for each single combination of all samples and the difference expressed as average ratio (AR). 2 way-ANOVA was calculated for the group-to-group study and proteins showing at least ± 1.5 AR (p < 0.05), were chosen for protein identification. The results for the response difference between WT and ∆*tmk1* to host versus self (WT-response *vs* ∆*tmk1-*response) were calculated by subtraction Log FC (fold change) base 2 of the response difference between WT (WT-response) and ∆*tmk1* (∆*tmk1-*response), where for each of them their host fungus-induced expressions were subtracted from growth under non-mycoparasitic conditions (self-confrontation control). Image preprocessing was carried out by Image Quant v7.0 and included only brightness and contrast adjustments for the respective color channel or equally to all color channels in the case of the DIGE experiment.

### In-gel tryptic digestion and protein identification

DIGE separated proteins were visualized by MS-compatible silver-staining and identified after in gel digestion carried out as previously described^59^. Briefly, spots of interest were excised, destained and incubated with 150 ng trypsin (proteomics grade, Roche, Basel, CH) in 50 mM NH_4_HCO_3_ containing 5% ACN. Digestion was carried out for 10 min at 170 W in a domestic microwave and subsequently overnight at 37 °C under mild agitation. Peptides were extracted, vacuum-dried, and stored at − 20 °C.

Protein identification was performed using a nanoLC-MS^n^ system (HCT, Bruker Daltonics, coupled with an Ultimate 3000, Dionex) using an Acclaim PepMap 100 column (375 µm OD/75 µm ID × 15 cm, 3 µm, 100 Å; ThermoFisher Scientific). Tryptic peptides were desalted (C18 ZipTip®, Millipore) before 1 µL sample dissolved in 0.05% formic acid (FA) containing 5% ACN was injected. Spectra were acquired between m/z 300–1500 over 90 min gradient elution at 250 nL/min. Automatic MS/MS-acquisition was set as follows: 1 precursor ion over 25 s, threshold relative 5%, active exclusion 0.25 min. Data processing was carried out by Data Analysis v3.2 and Biotools v3.2 (both Bruker Daltonics) and a Mascot v2.2.06 (http://www.matrixscience.com) in-house search engine using a FASTA file compiled from a total of 84,102,370 sequences, 182,549 sequences specific for *Trichoderma* species. Search parameters: taxonomy fungi, precursor ion mass tolerance 0.5 Da, fragment ion tolerance 0.5 Da, monoisotopic masses, charge state 1+ , 2+ and 3+, one missed cleavage, fixed modification carboxyamidomethylation, variable modification methionine oxidations.

### Phosphoproteome analysis

#### Comparative SDS-PAGE

Protein precipitates of three different samples from confrontations assays were used for comparison, *T. atroviride* WT and ∆*tmk1* mutant against *R.* *solani* and *T.* *atroviride* WT samples from self-confrontations. 32 µg protein of each sample were separated on precast 4–20% Tris-Glycin gels (details see SDS PAGE), visualized by MS-compatible Coomassie Brilliant Blue R250 staining and each gel lane was cut into 14 pieces according to given protein pattern. Gel pieces were subjected to in-gel tryptic digestion as described above. Peptides were desalted on HyperSep-96 C18 well plates (Thermo Scientific) and phosphopeptides were subsequently enriched with a Magnetic Titanium Dioxide Phosphopeptide kit (Pierce, Thermo Fisher). Phosphopeptides were eluted from the beads according to the manufacturer’s protocol and analyzed by MALDI-MS on an ultrafleXtreme (Bruker, Bremen,D) using a-cyano-4-hydroxy cinnamic acid as matrix (3 mg/mL in water/ACN 3/7), positive ion mode, and LIFT experiments for MS/MS analysis of at least three peptides for protein identification using the same routine as described above (charge state was 1+ only).

#### Shotgun analysis

Samples from confrontation assays of *T. atroviride* WT against *R. solani* were used for a global shotgun approach as active signaling pathways are expected to overexpress phosphoproteins. Protein precipitates (2 mg and 10 mg total protein) were dissolved in 500 µL 6 M urea/2 M thiourea solution at pH 8.5. After reduction with 50 µL 400 mM DTT and alkylation with 50 µL 800 mM IAA-solution the samples were digested over night at 37 °C with 500 µg trypsin (Sigma) in 50 mM NH_4_HCO_3_. After desalting with a Strata™-X 33 µm Polymeric RP column, peptides were loaded onto TiO_2_ beads (GL Sciences, Tokyo, J) (7/1 TiO_2_/peptide conc.) in a buffer containing 80% ACN, 5% TFA and 15% v/v lactic acid. After 15 min incubation, beads were washed with loading puffer, 80% aqueous ACN (0.1% TFA) and 20% aqueous ACN (0.1% TFA). Beads were dried in the vacuum centrifuge for 10 min, mixed with NH_4_OH (pH 11) and incubated for 15 min. The supernatant was separated, pH-adjusted with formic acid (FA, pH 5) and again desalted with an Empore™ C18-SD column. Samples were evaporated to dryness and a portion equivalent to 10 µg peptide was dissolved in buffer A (2% ACN, 98% water, 0.1% FA) for protein identification by LC–ESI–MS/MS analysis using a µ-Precolumn™ (300 µm × 5 mm) filled with PepMap™ C18 (5 µm, 100 Å) in combination with a NanoEase™ column (7 µm × 15 cm) filled with Atlantis™ dC18 (3 µm) material. Peptides were separated over 60 min by gradient elution (gradient from 1 to 98% aqueous ACN containing 0.1% FA, flow rate 300 nL/min). The LC system was connected to a Q-TOF Ultima™ API (AB Sciex, Framingham, MA, US). Data were analysed by MassLynx v4.1 and protein identification was carried out in Phenyx 2.1 using *T.atroviride* FrozenGeneCatalog_20100319 (11,863 sequences; 5,392,596 residues) as database. Samples were digested, enriched and analyzed multiple times and proteins were considered as identified if peptides were identified with significant scores (p < 0.05) in at least three out of five separations. All identified phosphopeptides were checked for meaningfulness against NetPhos v.2.

### Supplementary Information

Supplementary Tables.

Supplementary Figure S1.

## Data Availability

All data generated or analyzed during this study are included in this published article and its Supplementary information files. The datasets generated and/or analyzed during the current study are also available in the ArrayExpress repository [E-MTAB-13202].

## References

[1] Schaeffer HJ, Weber MJ (1999). Mitogen-activated protein kinases: Specific messages from ubiquitous messengers. Mol. Cell. Biol..

[2] Hamel L-P, Nicole M-C, Duplessis S, Ellis BE (2012). Mitogen-activated protein kinase signaling in plant-interacting fungi: Distinct messages from conserved messengers. Plant Cell.

[3] Krysan PJ, Colcombet J (2018). Cellular complexity in MAPK signaling in plants: Questions and emerging tools to answer them. Front. Plant Sci..

[4] Zhang W, Liu HT (2002). MAPK signal pathways in the regulation of cell proliferation in mammalian cells. Cell Res..

[5] Gustin MC, Albertyn J, Alexander M, Davenport K (1998). MAP kinase pathways in the yeast *Saccharomyces cerevisiae*. Microbiol. Mol. Biol. Rev..

[6] Rispail N (2009). Comparative genomics of MAP kinase and calcium-calcineurin signalling components in plant and human pathogenic fungi. Fungal Genet. Biol..

[7] Xu J-R (2000). MAP kinases in fungal pathogens. Fungal Genet. Biol..

[8] Zhao L-J (2007). Mitogen-activated protein kinase signalling pathways triggered by the hepatitis C virus envelope protein E2: Implications for the prevention of infection. Cell Prolif..

[9] Xu JR, Hamer JE (1996). MAP kinase and cAMP signaling regulate infection structure formation and pathogenic growth in the rice blast fungus *Magnaporthe grisea*. Genes Dev..

[10] Soanes DM, Chakrabarti A, Paszkiewicz KH, Dawe AL, Talbot NJ (2012). Genome-wide transcriptional profiling of appressorium development by the rice blast fungus *Magnaporthe oryzae*. PLoS Pathog..

[11] Jin Q (2013). Complexity of roles and regulation of the PMK1-MAPK pathway in mycelium development, conidiation and appressorium formation in *Magnaporthe oryzae*. Gene Expr. Patterns.

[12] Xiong D, Yu L, Shan H, Tian C (2021). *CcPmk1* is a regulator of pathogenicity in *Cytospora chrysosperma* and can be used as a potential target for disease control. Mol. Plant Pathol..

[13] Harman GE, Howell CR, Viterbo A, Chet I, Lorito M (2004). *Trichoderma* species-opportunistic, avirulent plant symbionts. Nat. Rev. Microbiol..

[14] Druzhinina IS (2011). *Trichoderma*: The genomics of opportunistic success. Nat. Rev. Microbiol..

[15] Atanasova L (2013). Comparative transcriptomics reveals different strategies of *Trichoderma* mycoparasitism. BMC Genom..

[16] Atanasova L (2018). The Gpr1-regulated Sur7 family protein Sfp2 is required for hyphal growth and cell wall stability in the mycoparasite *Trichoderma* atroviride. Sci. Rep..

[17] Kubicek CP (2011). Comparative genome sequence analysis underscores mycoparasitism as the ancestral life style of *Trichoderma*. Genome Biol..

[18] Reithner B (2007). Signaling via the *Trichoderma* atroviride mitogen-activated protein kinase Tmk 1 differentially affects mycoparasitism and plant protection. Fungal Genet. Biol..

[19] Soanes DM (2008). Comparative genome analysis of filamentous fungi reveals gene family expansions associated with fungal pathogenesis. PLoS ONE.

[20] Cullen PJ (2004). A signaling mucin at the head of the Cdc42- and MAPK-dependent filamentous growth pathway in yeast. Genes Dev..

[21] Qi S (2018). Cross-pathway control gene CPC1/GCN4 coordinates with histone acetyltransferase GCN5 to regulate catalase-3 expression under oxidative stress in *Neurospora crassa*. Free Radic. Biol. Med..

[22] Zhu X, Soliman A, Islam MR, Adam LR, Daayf F (2017). *Verticillium dahliae’s* isochorismatase hydrolase is a virulence factor that contributes to interference with potato’s salicylate and jasmonate defense signaling. Front. Plant Sci..

[23] Lafon A, Han K-H, Seo J-A, Yu J-H, d’Enfert C (2006). G-protein and cAMP-mediated signaling in aspergilli: A genomic perspective. Fungal Genet. Biol..

[24] Jedd G, Chua N-H (2000). A new self-assembled peroxisomal vesicle required for efficient resealing of the plasma membrane. Nat. Cell Biol..

[25] Moon AL, Janmey PA, Louie KA, Drubin DG (1993). Cofilin is an essential component of the yeast cortical cytoskeleton. J. Cell Biol..

[26] Egan MJ, McClintock MA, Reck-Peterson SL (2012). Microtubule-based transport in filamentous fungi. Curr. Opin. Microbiol..

[27] Oh YT (2013). *Aspergillus nidulans* translationally controlled tumor protein has a role in the balance between asexual and sexual differentiation and normal hyphal branching. FEMS Microbiol. Lett..

[28] Kingsbury JM, Sen ND, Cardenas ME (2015). Branched-chain aminotransferases control TORC1 signaling in *Saccharomyces cerevisiae*. PLoS Genet..

[29] Druzhinina IS, Shelest E, Kubicek CP (2012). Novel traits of *Trichoderma* predicted through the analysis of its secretome. FEMS Microbiol. Lett..

[30] Schmoll M (2016). The genomes of three uneven siblings: Footprints of the lifestyles of three *trichoderma* species. Microbiol. Mol. Biol. Rev..

[31] Delgado-Jarana J, Sousa S, González F, Rey M, Llobell A (2006). ThHog1 controls the hyperosmotic stress response in *Trichoderma harzianum*. Microbiol. Read. Engl..

[32] Gruber S, Zeilinger S (2014). The transcription factor Ste12 mediates the regulatory role of the Tmk1 MAP kinase in mycoparasitism and vegetative hyphal fusion in the filamentous fungus *Trichoderma atroviride*. PLoS ONE.

[33] Kumar A (2010). Overlapping and distinct functions of two *Trichoderma virens* MAP kinases in cell-wall integrity, antagonistic properties and repression of conidiation. Biochem. Biophys. Res. Commun..

[34] Moreno-Ruiz D, Salzmann L, Fricker M, Zeilinger S, Lichius A (2021). Stress-activated protein kinase signalling regulates mycoparasitic hyphal-hyphal interactions in *Trichoderma atroviride*. J. Fungi.

[35] Mukherjee PK, Latha J, Hadar R, Horwitz BA (2003). TmkA, a mitogen-activated protein kinase of *Trichoderma virens*, is involved in biocontrol properties and repression of conidiation in the dark. Eukaryot. Cell.

[36] Son H (2012). Differential roles of pyruvate decarboxylase in aerial and embedded mycelia of the ascomycete *Gibberella zeae*. FEMS Microbiol. Lett..

[37] Howden AJM, Preston GM (2009). Nitrilase enzymes and their role in plant-microbe interactions. Microb. Biotechnol..

[38] Basile LJ, Willson RC, Sewell BT, Benedik MJ (2008). Genome mining of cyanide-degrading nitrilases from filamentous fungi. Appl. Microbiol. Biotechnol..

[39] Michelsen CF, Stougaard P (2012). Hydrogen cyanide synthesis and antifungal activity of the biocontrol strain *Pseudomonas fluorescens* In5 from Greenland is highly dependent on growth medium. Can. J. Microbiol..

[40] Garrett MD, Zahner JE, Cheney CM, Novick PJ (1994). GDI1 encodes a GDP dissociation inhibitor that plays an essential role in the yeast secretory pathway. EMBO J..

[41] Morano KA (2007). New tricks for an old dog: The evolving world of Hsp70. Ann. N. Y. Acad. Sci..

[42] Nguyen EV (2016). Quantitative site-specific phosphoproteomics of *Trichoderma reesei* signaling pathways upon induction of hydrolytic enzyme production. J. Proteome Res..

[43] McNally MT, Free SJ (1988). Isolation and characterization of a *Neurospora* glucose-repressible gene. Curr. Genet..

[44] Parsons JF, Calabrese K, Eisenstein E, Ladner JE (2003). Structure and mechanism of *Pseudomonas aeruginosa* PhzD, an isochorismatase from the phenazine biosynthetic pathway. Biochemistry (Mosc.).

[45] Gehring AM, Bradley KA, Walsh CT (1997). Enterobactin biosynthesis in *Escherichia coli*: Isochorismate lyase (EntB) is a bifunctional enzyme that is phosphopantetheinylated by EntD and then acylated by EntE using ATP and 2,3-dihydroxybenzoate. Biochemistry (Mosc.).

[46] Wang M (2014). Identification of the role of a MAP kinase Tmk2 in *Hypocrea jecorina* (*Trichoderma reesei*). Sci. Rep..

[47] Pennington K, Chan T, Torres M, Andersen J (2018). The dynamic and stress-adaptive signaling hub of 14-3-3: Emerging mechanisms of regulation and context-dependent protein–protein interactions. Oncogene.

[48] van Heusden GP (1995). The 14-3-3 proteins encoded by the BMH1 and BMH2 genes are essential in the yeast *Saccharomyces cerevisiae* and can be replaced by a plant homologue. Eur. J. Biochem..

[49] Maere S, Heymans K, Kuiper M (2005). BiNGO: A Cytoscape plugin to assess overrepresentation of gene ontology categories in biological networks. Bioinform. Oxf. Engl..

[50] Shannon P (2003). Cytoscape: A software environment for integrated models of biomolecular interaction networks. Genome Res..

[51] Reiner, D. J. Small GTPases. In *WormBook* 1–65. 10.1895/wormbook.1.67.2 (2018). 10.1895/wormbook.1.67.2 PMC6369420 27218782

[52] Warris A, Ballou ER (2019). Oxidative responses and fungal infection biology. Semin. Cell Dev. Biol..

[53] Zeilinger S (1999). Chitinase gene expression during mycoparasitic interaction of *Trichoderma harzianum* with its host. Fungal Genet. Biol..

[54] Ruepp A (2004). The FunCat, a functional annotation scheme for systematic classification of proteins from whole genomes. Nucleic Acids Res..

[55] Grigoriev IV (2014). MycoCosm portal: Gearing up for 1000 fungal genomes. Nucleic Acids Res..

[56] Krogh A, Larsson B, von Heijne G, Sonnhammer EL (2001). Predicting transmembrane protein topology with a hidden Markov model: Application to complete genomes. J. Mol. Biol..

[57] Petersen TN, Brunak S, von Heijne G, Nielsen H (2011). SignalP 4.0: Discriminating signal peptides from transmembrane regions. Nat. Methods.

[58] Almagro Armenteros JJ (2019). Detecting sequence signals in targeting peptides using deep learning. Life Sci. Alliance.

[59] Marchetti-Deschmann M, Kemptner J, Reichel C, Allmaier G (2009). Comparing standard and microwave assisted staining protocols for SDS-PAGE of glycoproteins followed by subsequent PMF with MALDI MS. J. Proteom..

