# Supplementary material for: Mycoparasitism related targets of Tmk1 indicate stimulating regulatory functions of this MAP kinase in Trichoderma atroviride
Source: Sci Rep. 2023 Nov 15;13:19976. doi: 10.1038/s41598-023-47027-6 (PMC10651915; doi:10.1038/s41598-023-47027-6)
Supplement: Supplementary file 2 — Supplementary Figure S1. [file 41598_2023_47027_MOESM2_ESM.pdf]

Mycoparasitism related targets of Tmk1 indicate stimulating regulatory functions of this MAP kinase in *Trichoderma atroviride*

Lea Atanasova[1,2$*], Martina Marchetti-Deschmann[3$], Albert Nemes[3], Bianca Bruckner[3], Pavel Rehulka[3,4], Nancy Stralis-Pavese[5], Paweł P. Łabaj[5], David P. Kreil[5*] and Susanne Zeilinger[2*]

Supplementary figure

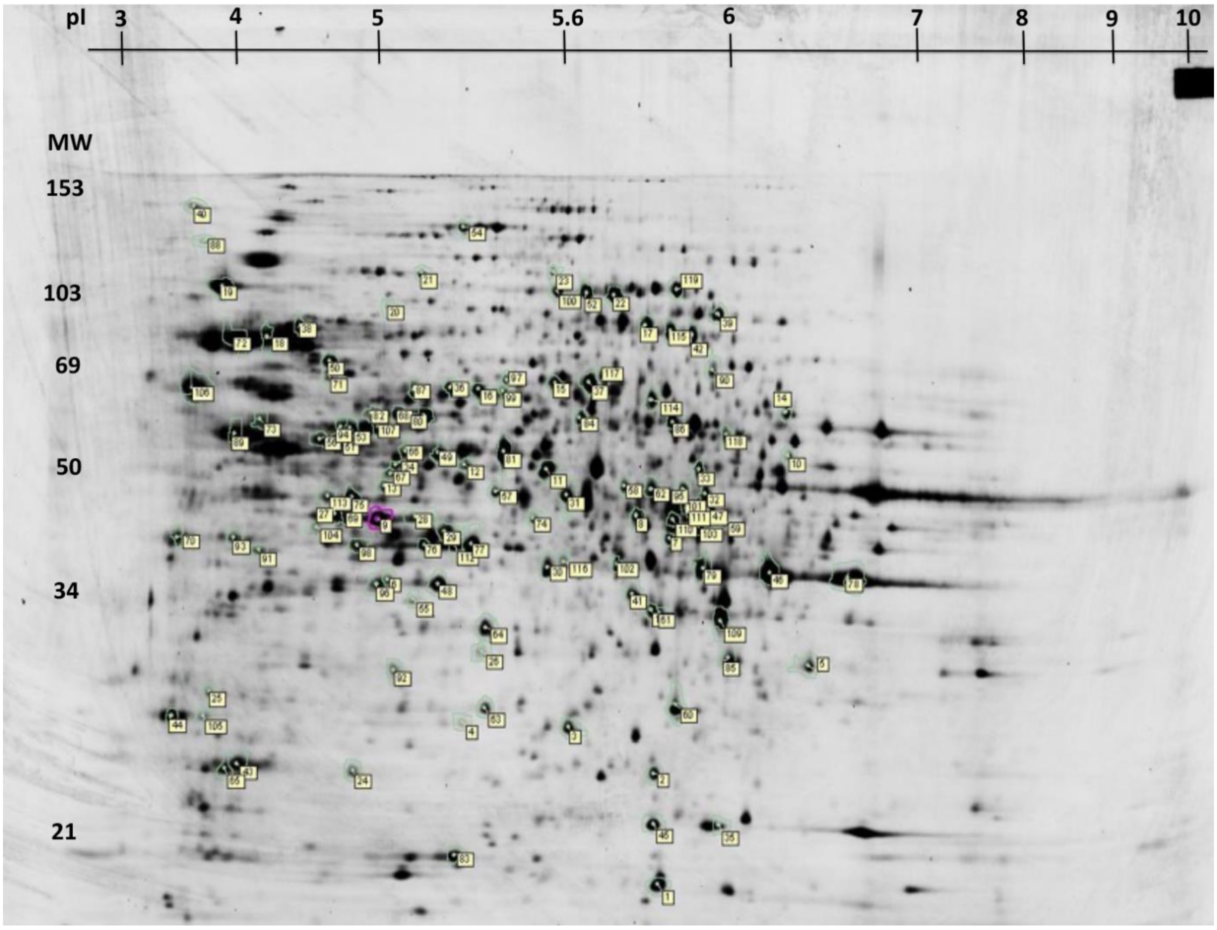

Figure S1: 2D master gel. The gel with the highest spot count from the DIGE approach including the technical replicates with spot numbers for proteins identified by LC-MS/MS analysis was assigned as master gel. Details of spot assignment are given in the materials and method part.
